# Retinoid X receptor agonist LG100268 modulates the immune microenvironment in preclinical breast cancer models

**DOI:** 10.1038/s41523-019-0135-5

**Published:** 2019-11-01

**Authors:** Ana S. Leal, Kayla Zydeck, Sarah Carapellucci, Lyndsey A. Reich, Di Zhang, Jessica A. Moerland, Michael B. Sporn, Karen T. Liby

**Affiliations:** 1 Department of Pharmacology & Toxicology, Michigan State University, East Lansing, MI USA; 2 Department of Molecular and Systems Biology, Dartmouth/Geisel School of Medicine at Dartmouth, Hanover, NH USA

**Keywords:** Breast cancer, Cancer microenvironment, Pharmaceutics, Tumour immunology

## Abstract

Despite numerous therapeutic advances in the past decade, breast cancer is expected to cause over 42,000 deaths in the United States in 2019. Breast cancer had been considered an immunologically silent tumor; however recent findings suggest that immune cells play important roles in tumor growth even in the breast. Retinoid X receptors (RXRs) are a subclass of nuclear receptors that act as ligand-dependent transcription factors that regulate a variety of cellular processes including proliferation and differentiation; in addition, they are essential for macrophage biology. Rexinoids are synthetic molecules that bind and activate RXRs. Bexarotene is the only rexinoid approved by the FDA for the treatment of refractory cutaneous T-cell lymphoma. Other more-potent rexinoids have been synthesized, such as LG100268 (LG268). Here, we report that treatment with LG 268, but not bexarotene, decreased infiltration of myeloid-derived suppressor cells and CD206-expressing macrophages, increased the expression of PD-L1 by 50%, and increased the ratio of CD8/CD4, CD25 T cells, which correlates with increased cytotoxic activity of CD8 T cells in tumors of MMTV-Neu mice (a model of HER2-positive breast cancer). In the MMTV-PyMT murine model of triple negative breast cancer, LG268 treatment of established tumors prolonged survival, and in combination with anti-PD-L1 antibodies, significantly (*p* = 0.05) increased the infiltration of cytotoxic CD8 T cells and apoptosis. Collectively, these data suggest that the use of LG268, a RXR agonist, can improve response to immune checkpoint blockade in HER2+ or triple-negative breast cancer.

## Introduction

Breast cancer is the most frequently diagnosed cancer in the United States of America, claiming over 42,000 lives each year.^1^ Numerous treatments have improved survival; however, aggressive subtypes, such as triple negative (TNBC) and epidermal growth factor receptor 2 (HER2)-positive breast cancer, are still challenging to treat and often deadly. Recognition of the influence of the tumor microenvironment, specifically the immune cell compartment, has gained in importance over the past decade and is now considered a hallmark of tumor progression and resistance to therapy.^2,3^ Infiltrating lymphocytes have prognostic properties clinically, specifically, lower levels of CD8 T cells in HER2 positive or TNBC, correlate with lower survival rates and response to therapy.^4^ In addition, macrophages were also shown to be essential for breast cancer progression, both in mouse models and human disease.^2,5,6^

Tumor cells utilize different strategies to escape immune surveillance, and one of the most effective is their expression of immune checkpoints. Immune checkpoints are essential for the regulation of normal immune system homeostasis, specifically the activation of T cells. Cancer cells and immune cells within the cancer microenvironment use the expression of inhibitory immune checkpoints to evade immune surveillance and consequently avoid elimination by the immune system.^7^ Programed death-1 (PD-1) is a transmembrane protein receptor, which functions as a major negative regulator that controls T-cell activation, T-cell exhaustion, T-cell tolerance, and resolution of inflammation.^8^ Expression of PD-1 ligand (PD-L1) can be increased by pro-inflammatory signals to serve as a negative feedback mechanism, decreasing T-cell activation and cytokine production.^9^ Several drugs that target inhibitory immune checkpoints, such as PD-1/PD-L1 and cytotoxic T-lymphocyte-associated protein 4, have been developed. The FDA approval of anti-PD-L1 antibodies (atezolizumab) in 2016 has opened a new therapeutic approach to treat cancer. Most breast cancer patients have not benefitted from immune checkpoint blockade because of conflicting results between studies and the lack of reliable biomarkers.^10^ However, a recent clinical trial showed increased progression-free survival in TNBC patients positive for PD-L1 who were treated with a combination of atezolizumab and nab-paclitaxel as first line therapy.^11^

**Fig. 1:**
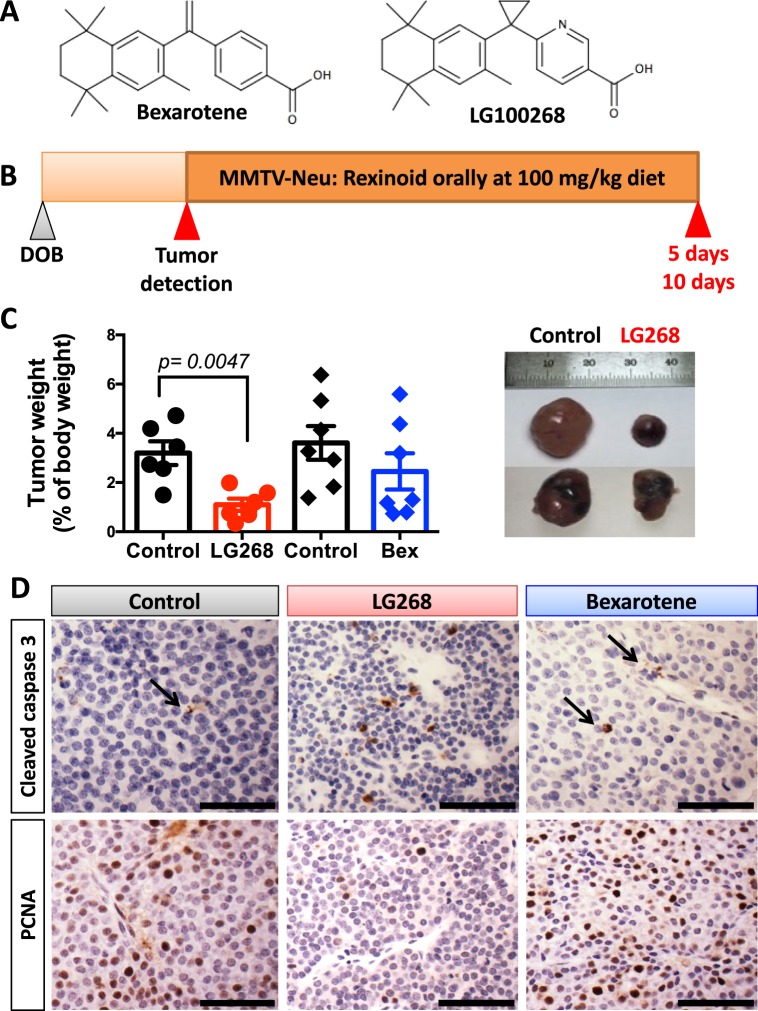
RXR agonist LG268 reduces tumor burden in MMTV-Neu mice. **a** Chemical structures for bexarotene and LG100268 (LG268). **b** Experimental design for the treatment of MMTV-Neu mice with rexinoids. MMTV-Neu mice with tumor(s) with a volume of 32–64 mm^3^ were treated with control diet or LG268 (100 mg/kg diet) for 5 days or with control diet and bexarotene (100 mg/kg diet) for 10 days. **c** Tumors were harvested at the end of the study and weighed. Tumor weight is shown as percentage of total body weight. *n* = 6–7 mice/group (*t* = 4.837; df = 5). Bars represent average and error bars the standard error of the mean (SEM). **d** Immunohistochemistry for cleaved caspase 3 and proliferating cell nuclear antigen (PCNA) in tumors. Arrows point to positive cleaved caspase 3 in control and bexarotene treated tumors. Scale bar represents 60 μm

The retinoid X receptor (RXR) is a member of the nuclear receptor superfamily of ligand-dependent transcription factors that bind various lipophilic hormones and lipid metabolites.^12,13^ RXR forms homodimers and heterodimers with many other members of this nuclear receptor superfamily, such as the peroxisome proliferator-activated receptor (PPAR), liver X receptor (LXR), retinoic acid receptor (RAR), and vitamin D receptor. RXR and its heterodimers have a profound effect on the function of myeloid cells and link cellular metabolism and immune function.^14,15^ Specific activation of RXR up regulates chemokine expression, promotes phagocytosis of apoptotic cells, and attenuates antiviral responses in myeloid cells.^14,16^ Bexarotene (Targretin, Fig. 1a), a synthetic RXR agonist, was approved by the FDA in 1999 for treatment of cutaneous T-cell lymphoma^17^ and has been tested clinically for treatment of lung and breast cancer.^18,19^ Several more potent RXR agonists have been synthesized, including LG100268 (LG268, Fig. 1a).^20,21^

We and others have shown that LG268 effectively prevents the development of tumors and treats established tumors in the MMTV-Neu mouse model of breast cancer; this rexinoid also increased apoptosis and decreased vascularization in the tumors.^22,23^ Although bexarotene also prevents the development of mammary tumors by reducing cell proliferation,^24,25^ it was ineffective in a clinical trial for treating metastatic breast cancer.^18^ Because of the connection between the RXR and myeloid cell functions,^14^ both LG268 and bexarotene would be expected to regulate immune functions, but the effects of these rexinoids on immune cells within a tumor remains mostly unexplored. Because (a) RXR regulates the myeloid cell compartment, (b) mouse models of breast cancer are dependent on macrophages to promote cancer,^2,26^ and (c) LG268 has little effect on proliferation of tumor cells in vitro; we hypothesized that LG268 has an immune modulatory effect in mammary gland tumors that contributes to the striking tumor regression previously observed in experimental breast cancer.^22,23^

Therefore, we used two preclinical mouse models of breast cancer: MMTV-Neu, which replicates HER2-positive breast cancer and MMTV-PyMT transgenic mice, an aggressive autochthonous model of triple negative breast cancer (mice do not express estrogen, progesterone, or HER2 receptors).^27–29^ In MMTV-Neu mice, unactivated Neu (ErbB2/HER2) protein expression is targeted to the mammary gland by a MMTV promoter.^27^ ErbB2/HER2 is a tyrosine kinase receptor that promotes tumor growth, and this protein is overexpressed in 20–30% of breast cancer patients. Patients that test positive for ErbB2/HER2 receive trastuzumab (Herceptin), a targeted therapy. MMTV-Neu mice respond to trastuzumab, confirming the relevance of the model for studying ErB2/HER2-positive breast cancer.^27^ Expression of the polyoma middle T oncoprotein (PyMT) in the mammary epithelium drives the formation of aggressive tumors in the mammary gland of mice.^28^ These highly aggressive tumors do not express progesterone, estrogen, or HER2 receptors and are unresponsive to conventional therapies, mirroring human TNBC.^30^

Here, we show that LG268, but not bexarotene, exerts significant immunomodulatory effects in MMTV-Neu and PyMT mouse models of HER2-positive and TNBC breast cancer models, respectively. Moreover, LG268 increases both expression of PD-L1 in macrophages and tumor-suppressive immune cell populations within the tumor microenvironment, creating a favorable milieu for the combination of appropriate RXR agonists with anti-PDL-1 antibodies.

## Results

### LG268 reduced tumor burden in MMTV-Neu mice treated for only 5 days

We have previously shown that the rexinoid LG268 reduces tumor burden and induces regression of established tumors in the MMTV-Neu model, by decreasing cell proliferation and inducing apoptosis.^22,23^ Moreover, bexarotene prevents the development of tumors in mammary glands.^24^ Despite the promising efficacy in this model, the mechanism of action for the RXR agonists LG268 and bexarotene remain unknown, mostly because these small molecules fail to induce tumor cell death in vitro. Based on these observations, we tested whether the RXR agonist LG268 induces tumor regression through an immunologically dependent mechanism. Experimentally, a short treatment period with rexinoids was chosen to allow the collection of tissues for analysis, as previous studies demonstrated that LG268 can induce total regression of tumors.^22^ Bexarotene, the only FDA-approved RXR agonist, was also used for comparison to LG268.

Mice with mammary tumors at least 5 × 5 mm in size were treated for 5 days with LG268 or for 10 days with bexarotene. Both rexinoid treatments were 100 mg/kg of diet, which is equivalent to 25 mg/kg of body weight (Fig. 1b). In only 5 days, treatment with LG268 significantly (*p* = 0.0047) reduced the tumor size from 3.2% of total body weight in control diet to 1.1% of total body weight (Fig. 1c). Treatment with bexarotene did not significantly reduce the volume of the treated tumors, even after 10 days of treatment, when compared with control mice (Fig. 1c), even though four treated mice showed a partial response. The reduction in tumor size was accompanied by an increase in the expression of cleaved caspase 3 and a decrease in proliferating cell nuclear antigen (PCNA) (Fig. 1d), demonstrating the ability of LG268 to induce apoptosis (cleaved caspase 3) and reduce proliferation (PCNA). The presence of cleaved caspase 3 suggests an immunologically mediated cell death.^31^ Bexarotene did not increase caspase 3 cleavage or decrease PCNA in the tumors, consistent with the lack of reduction in tumor volume (Fig. 1d).

### Altered immune cell populations in MMTV-Neu mice treated with LG268

**Fig. 2:**
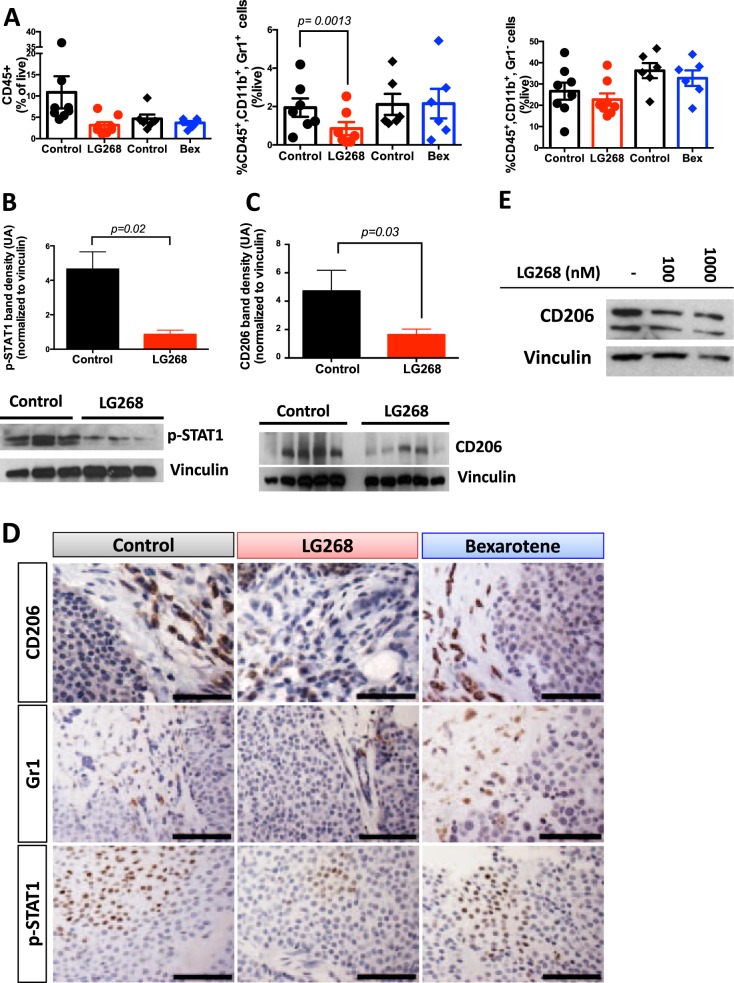
Treatment with LG268 reduces the infiltration of myeloid suppressor cells and CD206 expression in tumors from MMTV-Neu mice. MMTV-Neu mice with established tumors were treated with control diet or LG268 (100 mg/kg diet) for 5 days or with bexarotene (100 mg/kg diet) for 10 days. **a** Myeloid cell populations were labeled with appropriate antibodies and analyzed by flow cytometry: total immune cells with CD45^+^; macrophages were CD45^+^, CD11b^+^, Gr1^−^; and myeloid-derived suppressor cells were CD45^+^, CD11b^+^, Gr1^+^ (*n* = 6–7 mice/group; for MDSC: *t* = 5.625; df = 6). Levels of p-STAT1 (*t* = 3.688; df = 8) **b** and CD206 (*t* = 2.555; df = 10) **c** were evaluated by western blotting in tumor lysates (*n* = 9–11 mice/group). Quantification of the whole lysates is shown in the bar graphs, and a representative immunoblot is also shown. **d** Immunohistochemistry was used to confirm the levels of CD206, Gr1 and p-STAT1 in tumor sections. Scale bar represents 60 μm. **e** RAW cells stimulated with conditioned media from E18-14C-27 cells established from mammary tumors of MMTV-Neu mice were treated with LG268 (0–1000 nm) for 24 h and CD206 expression analyzed by western blotting. Bars represent average and error bars the standard error of the mean (SEM)

RXR and partner receptors play important roles in the biology of macrophages and other immune cells.^14^ Because breast cancer progression is highly dependent on myeloid cell populations, specifically macrophages,^2,5,6^ we assessed immune cell populations (gating strategy for flow cytometry shown in Supplementary Fig. 1) and the state of activation of these populations in the tumors of MMTV-Neu mice. Myeloid-derived suppressor cells (MDSCs; CD45^+^, CD11b^+^, Gr1^+^) are an important immune suppressive population in breast cancer.^32,33^ The RXR agonist LG268 decreased the percentage of MDSCs in the mammary gland tumors of MMTV-Neu mice (*p* = 0.0013), from 1.9 ± 0.5% of CD45^+^ immune cells in the control group to 0.8 ± 0.3% in the LG268-treated group (Fig. 2a, d). Notably, bexarotene treatment did not alter the percentage of infiltrating MDSCs compared with the control group in MMTV-Neu mice (Fig. 2a, d). The immunosuppressive phenotype of MDSCs is associated with the expression of p-STAT1.^34,35^ Treatment with LG268 significantly (*p* = 0.02) reduced the expression of p-STAT1 by 80% in whole tumor lysates, as observed by western blotting (Fig. 2b) and confirmed by immunohistochemistry (Fig. 2d). In contrast, no changes were observed in the levels of p-STAT1 in mice treated with bexarotene (Fig. 2d, Supplementary Fig. 2). No changes in the expression of p-STAT3 or p-STAT5 were observed in whole tumor lysates from these experiments (Supplementary Fig. 3).

Although the percentage of macrophages (CD45^+^, CD11b^+^, Gr1^−^) infiltrating into the tumors was not different between groups (Fig. 2a), treatment with LG268 significantly (*p* = 0.03) reduced the expression of CD206 by 65%, as evaluated by western blot in tumor lysates and immunohistochemistry of tumor sections (Fig. 2c, d). CD206 is a marker of tumor-promoting macrophages; in breast cancer, CD206+ macrophages contribute to tumor immunosuppression, angiogenesis, metastasis, and relapse.^36–38^ In contrast, bexarotene did not induce a change in the levels of CD206 in the tumors (Supplementary Fig. 2). No differences in the total number of immune cells (CD45^+^) infiltrating into the tumors (Fig. 2a) or changes in these immune cell populations in either the spleen or the intact mammary glands (Supplementary Fig. 4) were observed in any of the groups.

**Fig. 3:**
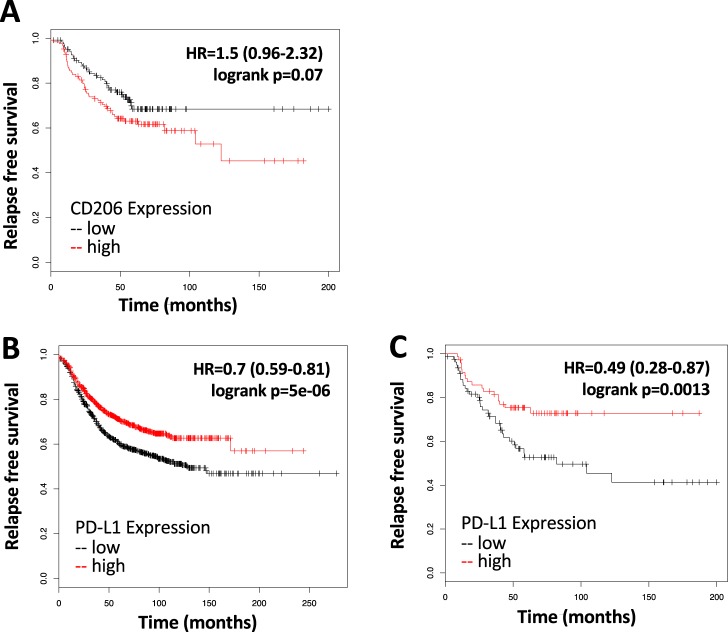
Prognostic impact of expression of CD206 and PD-L1 in breast cancer patients. Data were generated by accessing KMPlot (http://www.kmplot.com) and using the terms shown for each graph; no stratification strategy was used, except for analyzing the cohort of patients with HER2-positive breast cancer (*n* = 252; 126 low, 126 high in **a**). Prognostic of CD206 levels **a** in HER2-positive breast cancer patients, PD-L1 expression **b** in all breast cancer patients (*n* = 1764; 888 low, 876 high in **b**); or in HER2 positive breast cancer patients **c** (*n* = 150; low 79, high 71 in **c**)

To explore if the expression of CD206 in macrophages is dependent on stimulatory factors from tumor cells (tumor educated macrophages),^39^ conditioned media was collected from an established cell line (E18-14C-27 cells) derived from a mammary tumor of an MMTV-Neu mouse.^24^ When RAW 264.7 (macrophage-like) cells were stimulated with conditioned media from the E18-14C-27 cancer cells and treated with increasing concentrations of LG268 for 24 h, a decrease in the expression of CD206 was observed by immunoblotting (Fig. 2e). Confirming previous studies^23^ and the known role of tumor educated macrophages in tumor angiogenesis,^40^ treatment with LG268 reduced the caliber of the vessels in the tumors, as observed by CD31 immunohistochemistry (Supplementary Fig. 5). Notably, expression of CD206 predicts relapse-free survival in a meta-analysis^41^ of patients with HER2 positive breast cancer, irrespective of tumor grade or lymph node status (Fig. 3a).

**Fig. 4:**
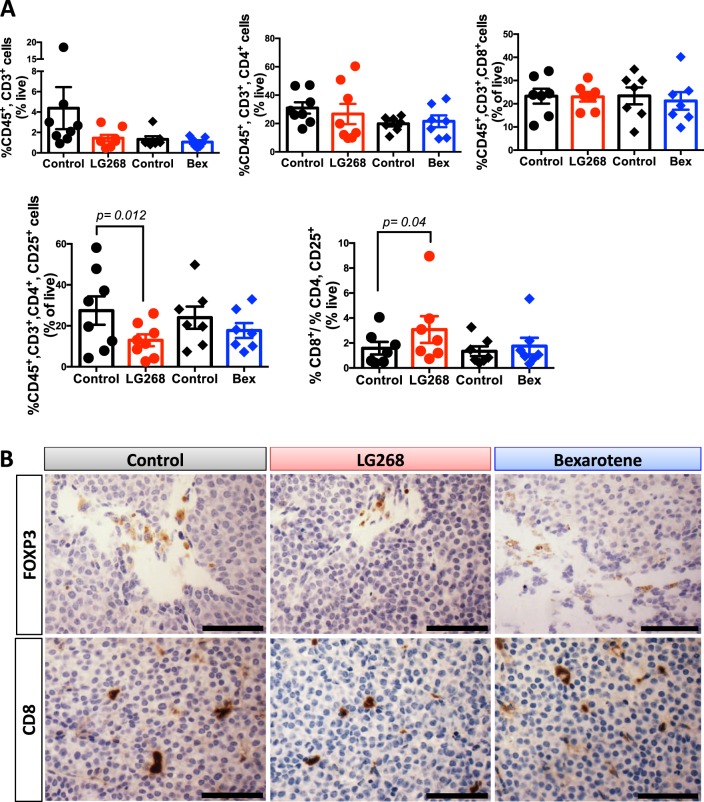
LG268 treatment modifies T-cell populations in established tumors. **a** T-cell populations in mammary gland tumors from MMTV-Neu mice treated with rexinoids (100 mg/kg diet) were analyzed by flow cytometry (*n* = 6–8 mice/group). Total T cells: CD45^+^, CD3^+^; CD4 T cells: CD45^+^, CD3^+^, CD4^+^; activated CD4 T cells: CD45^+^, CD3^+^, CD4^+^, CD25^+^ (*t* = 3.341; df = 7); CD8 T cells: CD45^+^, CD3^+^, CD8^+^. (ratio %CD8/%CD4, CD25: *t* = 2.543; df = 6). **b** Immunostaining of FOXP3 in tumor sections was used to confirm the downregulation of activated CD4 T cells observed by flow cytometry. Scale bar represents 60 μm. *n* = 7–8, bars represent average and error bars the standard error of the mean (SEM)

The immune tumor microenvironment is highly complex with numerous interactions.^42^ Various reports show cross-talk between myeloid cells and T cells.^43,44^ Because LG268 modulated the myeloid compartment in the tumors of MMTV-Neu mice (Fig. 2), we also analyzed the T-cell populations in these tumors. Treatment with LG268 significantly (*p* = 0.012) reduced the percentage of activated CD4 T cells (CD45^+^, CD3^+^, CD4^+^, CD25^+^) in the tumors compared with controls (27.5 ± 6.9% vs. 13.0 ± 2.9%, Fig. 4a). Activated CD4 T cells correlate with CD4 T cells expressing FOXP3 (Tregs) in human tumors, and high expression of FOXP3 is associated with reduced survival.^45^ Immunohistochemistry of the tumors from MMTV-Neu mice revealed that treatment with LG268 decreased the expression of FOXP3 (Fig. 4b), in agreement with the reduced infiltration of CD45^+^, CD3^+^, CD4^+^, CD25^+^-activated T cells observed by flow cytometry. Moreover, in the cohort of mice treated with LG268, tumors had a significantly (*p* = 0.04) higher ratio (3.1 ± 0.5, Fig. 4a) of CD8/CD4, CD25 vs. controls (1.6 ± 1.1). In contrast, no differences were observed in this ratio in the cohort of mice treated with bexarotene vs. control (Fig. 4a). A high ratio of CD8/CD4, CD25 cells is predictive of lower lymph nodes metastasis and overall increased survival in cancer patients.^45^ No alterations in the percentage of total T cells (CD45^+^, CD3^+^), CD4 (CD45^+^, CD3^+^, CD4^+^), or CD8 (CD45^+^, CD3^+^, CD8^+^) T cells were observed in the tumors (Fig. 4a) or in the spleen and intact mammary glands (Supplementary Fig. 6).

**Fig. 5:**
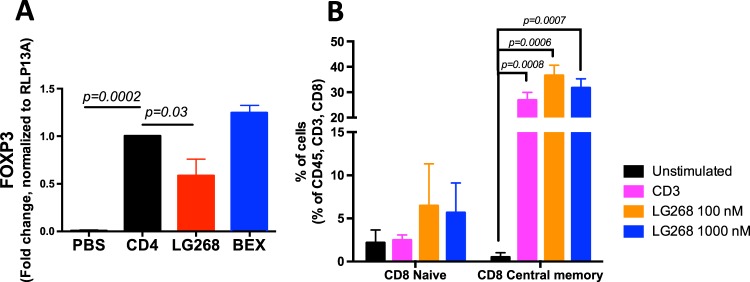
LG268 reduces the expression of FOXP3 in CD4 T cells and modulates ratios of CD8 T-cell populations in vitro. **a** CD4 T cells were isolated from a spleen of a wild-type mouse using negative magnetic beads. CD4 T cells were plated with anti-CD3, anti-CD28, IL2 and TGFβ for 24 h prior to adding LG268 or bexarotene for 4 days. CD4 cells where collected and levels of FOXP3 were determined by PCR. **b** CD3 cells were isolated with negative magnetic beads from the spleen of a wild-type mouse. CD3 T cells were stimulated with anti-CD3 and treated with LG268 for 3 days. Activation of CD4 and CD8 was evaluated by flow cytometry. Cells were stained with surface markers to identify different cell populations; Naïve: CD3^+^, CD8^+^, CD44^−^, CD62L^+^; Central memory: CD3^+^, CD8^+^, CD44^+^, CD62L^+^; Effector/effector memory: CD3^+^, CD8^+^, CD44^+^, CD62L^−^. *n* = 3–4, Bars represent average and error bars the standard error of the mean (SEM)

To interrogate the direct effects of LG268 on T cells, CD4 and CD3 T cells were isolated from normal murine spleens using negatively activated magnetic cell sorting (MACS) for in vitro assays. Isolated CD4 T cells were stimulated with anti-CD3ε, anti-CD28, IL2, and TGFβ for 24 h to skew towards regulatory T cells (Tregs). LG268 was then added and CD4 T cells were cultured for an additional 4 days. The RXR agonist LG268 reduced FOXP3 mRNA expression by 40% *(p* = 0.03*)* when compared with the vehicle control (Fig. 5a). Isolated CD3 T cells cultured in the presence of anti-CD3ε and LG268 also showed an increased percentage of CD8 naive and central memory cells (Fig. 5b). CD8 naive T-cell population are more effective at killing tumor cells than other CD8 subpopulations.^46^

### LG268 increased expression of the immune checkpoint PD-L1 and decreased PD-1 expression in MMTV-Neu mice

Although RXR agonists regulate several aspects of macrophage biology,^14^ their role in PD-1/PD-L1 regulation is not known. Elevated levels of PD-L1 enhance the response to treatment with PD-L1 inhibitors.^47^ Breast cancer patients with increased lymphocytic infiltrates and elevated levels of PD-L1 have increased survival expectancy.^48^ A meta-analysis^41^ of all breast cancer patients without stratification strategies reveled increased probability of relapse-free survival for patients with higher expression of PD-L1 (Fig. 3b). In addition, when the same meta-analysis^41^ is performed using data from only HER2-positive breast cancer patients, an augmented expression of PD-L1 is predictive of an elevated probability for relapse-free survival (Fig. 3c).

**Fig. 6:**
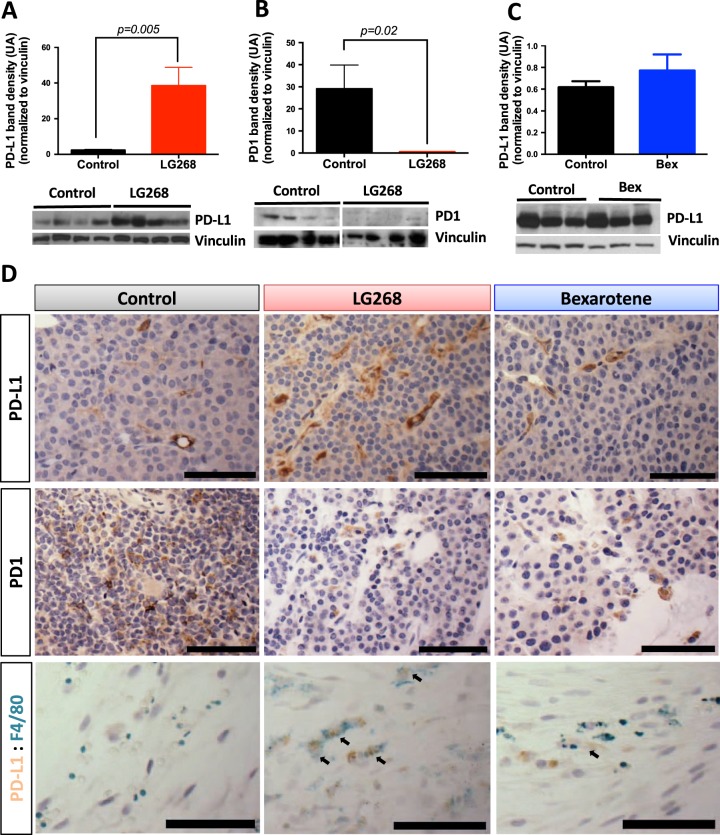
LG268 increases PD-L1 protein expression in tumors. **a** MMTV-Neu mice were treated with control diet or rexinoids (100 mg/kg diet). Levels of PD-L1 (*t* = 3.558; df = 10) (**a** and **c**) and PD-1 (*t* = 2.662; df = 10) **b** were analyzed by western blot in tumor lysates. Quantification of total protein levels in all samples (*n* = 11/group) is shown in the bar graphs, and western blots of representative samples are shown. **d** Immunohistochemistry was used to stain tumor sections for PD-L1 and PD-1, and dual immunohistochemical staining was used to stain tumor sections for PD-L1 and F4/80 (macrophages). Scale bar represents 60 μm. *n* = 5–11, Bars represent average and error bars the standard error of the mean (SEM)

**Fig. 7:**
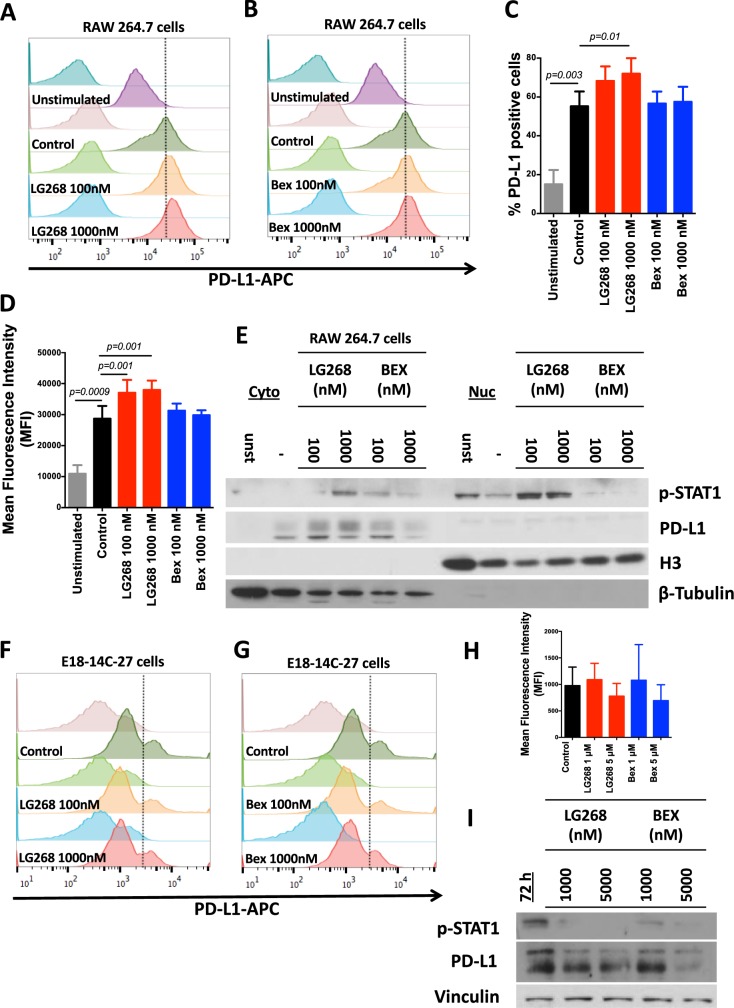
LG268 treatment increases the expression levels of PD-L1 in macrophages. RAW 264.7 macrophage-like cells were treated with conditioned media from E18-14C-27 breast cancer cells isolated from a mammary tumor in a MMTV-Neu mouse; representative histograms for levels of PD-L1 after treatment with LG268 **a** or bexarotene **b** for 24 h, are shown. Quantification of the percentage of positive PD-L1 (*F* = 7.74; df = 5) **c** or mean florescence intensity (*F* = 9.765; df = 5) **d** for four independent experiments. **e** Cytoplasmic (Cyto) vs. nuclear (Nuc) expression of PD-L1 and p-STAT1 in RAW 264.7 macrophage-like cells after stimulation with conditioned media from E18-14C-27 cancer cells. Representative histograms of PD-L1 expression in E18-14C-27 cancer cells after treatment with LG268 **f** or bexarotene **g** for 72 h. **h** Mean florescence intensity of E18-14C-27 cancer cells positive for PD-L1. **i** E18-14C-27 cancer cells were treated with LG268 or bexarotene for 72 h and levels of PD-L1 and p-STAT1 were analyzed by western blot. *n* = 3–4, Bars represent average and error bars the standard error of the mean (SEM)

Because mice treated with LG268 showed a favorable immune profile in the tumors, we interrogated the expression of PD-L1 and PD-1 within these tumors. Tumors treated with LG268 have significantly higher PD-L1 (*p* = 0.005) but lower PD-1 (*p* = 0.02) expression compared with control mice, as determined by western blots of total tumor lysates and by immunohistochemistry of tumor sections (Fig. 6a, b and d). Bexarotene failed to induce PD-L1 expression in the tumors of MMTV-Neu mice treated for 10 days (Fig. 6c, d). Co-staining with F4-80 and PD-L1 revealed that the expression of PD-L1 observed in the tumors of MMTV-Neu mice is mostly on infiltrating macrophages (Fig. 6d, Supplementary Fig. 7A). Additionally, PD-L1 expression was confirmed in CD11b positive cells isolated from the tumors of MMTV-Neu mice using positive activated MACS (Supplementary Fig. 7B). To confirm these changes in vitro, E18-14C-27 and RAW 264.7 cell lines were treated with LG268. RAW 264.7 cells stimulated with conditioned media from E18-14C-27C cells (tumor educated macrophages), showed significantly (*p* = 0.01 control vs. LG268 1000 nm) increased levels of PD-L1 upon treatment with LG268 at 1000 nm (control 55.2 ± 7.6% vs. LG268 1000 nm 72.0 ± 8.0%), but not with bexarotene (Fig. 7a–d). Similar observations were made in human THP-1 monocytic leukemia cells (surrogate macrophages) stimulated with conditioned media from human HER2-positive SK-BR-3 breast cancer cells and treated with LG268 or bexarotene for 24 h (Supplementary Fig. 7C, D). Because the PD-L1 promoter region has a binding site for p-STAT1,^49^ we interrogated if the increase in PD-L1 expression was owing to increased nuclear localization of p-STAT1. LG268, but not bexarotene, increased levels of p-STAT1 in the nucleus of RAW 264.7 cells stimulated with conditioned media from E18-14C-27 cells (Fig. 7e). The elevated nuclear localization of p-STAT1 was accompanied by increased cytoplasmic expression of PD-L1 (Fig. 7e). To investigate the effects of rexinoid treatment on PD-L1 expression in cancer cells, E18-14C-27 cells were treated with LG268 and bexarotene at 1 and 5 μm for 72 h. These higher concentrations did not induce cell death, and the levels of PD-L1 expression were slightly, but not significantly, reduced in the cancer cells (Fig. 7f–i). However, the expression of p-STAT1 was reduced in the E18-14C-27 cancer cells treated with rexinoids (Fig. 7i).

### LG268 extended survival of PyMT mice

**Fig. 8:**
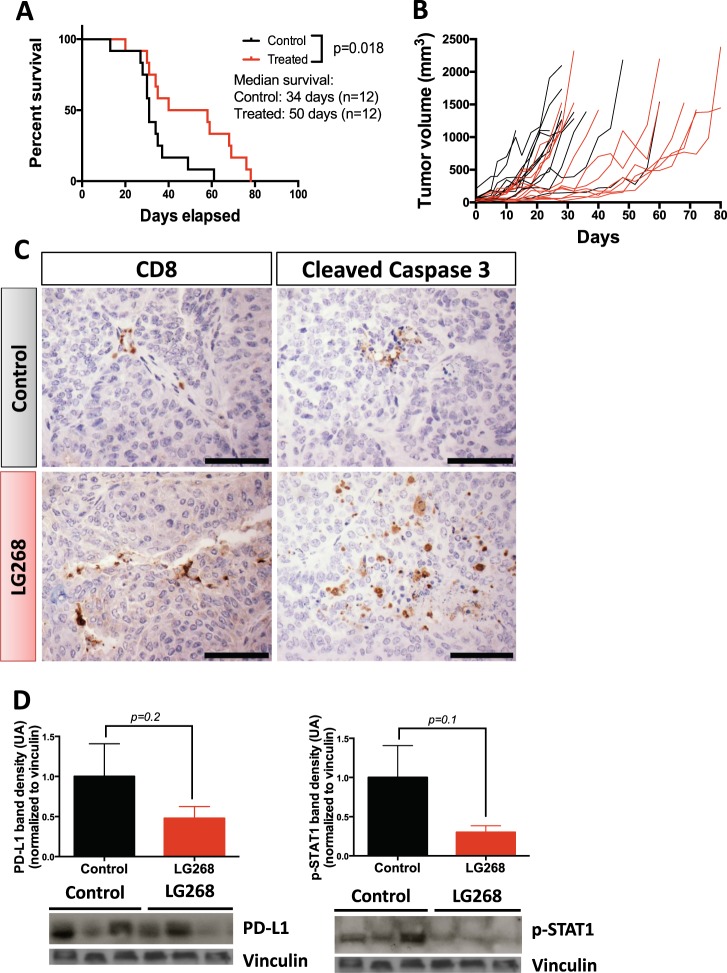
LG268 treatment prolongs survival in PyMT mice. **a** PyMT mice with tumor(s) 32–64 mm^3^ in size were treated with control diet or diet containing LG268 (100 mg/kg diet). Mice were kept on treatment until tumor burden reached IACUC-defined sizes (*n* = 12 mice/group). **b** Tumors were measured twice weekly with a caliper; total tumor volume was graphed over time. **c** Levels of cleaved caspase 3 and CD8 were determined by immunohistochemistry. Representative pictures for each group are shown. Scale bar represents 60 μm. **d** Levels of p-STAT1 and PD-L1 in tumor extract at the end point. Levels of the indicated proteins were determined by western blot. Western blot for four mice per cohort are represented. *n* = 12, bars represent average and error bars the standard error of the mean (SEM)

To determine whether the immune modulation induced by LG268 could extend survival in a model of TNBC, PyMT mice with tumor volumes of 32–64 mm^3^ (ranging in age from 15 to 27 weeks) were treated with LG268 (100 mg/kg of diet) or control diet until tumor burden met maximum defined endpoints (individual tumor volume < 520 mm^3^) allowed by IACUC. In control mice, tumors progressed extremely rapidly, with maximum tumor volume reached, on average, at 34 ± 3.4 days. Mice fed LG268 survived on average 50 ± 5.9 days, a significant (*p* = 0.018) extension compared with controls (Fig. 8a). Following tumor progression in each individual mouse over time showed that the volume of tumors in mice fed LG268 diet stabilized for 20–30 days, escaped, and then progressed rapidly (Fig. 8b). Following total volume of control vs. treated mice showed that increases in volume occurred faster in control than in treated groups, with significant (*p* < 0.05) differences between groups until day 31 (Supplementary Fig. 8A). The differences between groups then disappeared, as control mice reached the maximum allowed tumor burden and a reduced number of mice was not sufficient to detect statistically significant changes. There was no difference in total weight of the tumors at the time of necropsy, confirming that all mice were maintained in the study until disease burden was similar (Supplementary Fig. 8B).

Histological analysis of the tumors from LG268 fed mice showed higher number of cells undergoing apoptosis, as seen by immunohistochemistry for cleaved caspase 3 (Fig. 8c). In addition, an increased infiltration of CD8 T cells was found in PyMT mice treated with LG268. CD8 T cells can be observed in close contact with the tumor cells (Fig. 8c). A tendency for lower levels of PD-L1 and p-STAT1 were observed in whole lysates of tumors, determined by western blot (Fig. 8d). No differences were observed in the levels of CD206 and p-STAT3 in whole tumor lysates (Supplementary Fig. 9A).

### Combination of LG268 with anti-PD-L1 antibodies increased infiltration of cytotoxic CD8 T cells

**Fig. 9:**
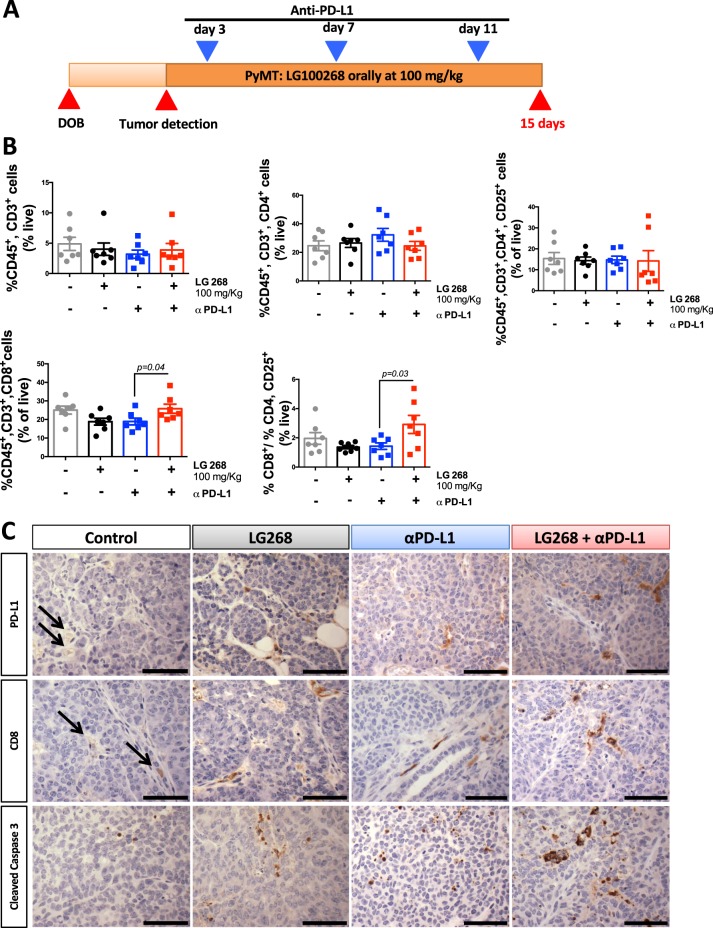
The combination of LG268 and anti-PD-L1 antibodies increases the infiltration of CD8 T cells and caspase 3 activation. **a** Experimental design. PyMT mice with tumor(s) 32–64 mm^3^ in size were started on control diet or LG268 diet (100 mg/kg) 48 h before the first anti-PD-L1 treatment. Anti-PD-L1 antibodies were injected i.p. (three injections each of 40 µg per mouse). Mice were killed on day 15. (*n* = 7 mice/group). **b** Immune cell infiltration in tumors was analyzed by flow cytometry. Total T cells: CD45^+^, CD3^+^; CD4 T cells: CD45^+^, CD3^+^, CD4^+^; activated CD4 T cells: CD45^+^, CD3^+^, CD4^+^, CD25^+^; CD8 T cells: CD45^+^, CD3^+^, CD8^+^ (*t* = 2.219; df = 12) (ratio %CD8/%CD4, CD25: *t* = 2.268; df = 12). **c** Immunohistochemistry for PD-L1, CD8 and cleaved caspase 3 in tumors. Scale bar represents 60 μm. *n* = 5–7, bars represent average and error bars the standard error of the mean (SEM)

When tumor burden met maximum defined endpoints allowed by the IACUC (end point), the levels of PD-L1 were not significantly different in tumors of PyMT mice treated with LG268 vs. control fed mice (Fig. 8d). Nevertheless, CD8 infiltration was increased in the mice treated with LG268 (Fig. 8c) so we interrogated if the tendency for lower PD-L1 levels was owing to resistance/escape from the immune modulatory effects of LG268. Experimentally, PyMT mice with a tumor volume of 32–64 mm^3^ were treated with LG268 (100 mg/kg of diet) or control diet for 14 days (Fig. 9a). Mice were killed at day 15, and tumor, intact mammary gland and spleen were analyzed by flow cytometry, immunohistochemistry, and immunoblotting (whole tumor lysates). At day 15, PyMT mice treated with LG268 had higher levels of PD-L1 in tumor lysates when compared with controls (Supplementary Fig. 9B). However, the LG268 treatment alone did not change any of the immune cell populations infiltrating into the tumors at day 15 (Fig. 9b).

Because elevated levels of PD-L1 in breast cancer patients correlates with increased survival (Fig. 3b, c) and increased likelihood of response to a PD-L1 immune checkpoint blockade,^47^ we hypothesized that the combination of an immune checkpoint inhibitor and LG268 would further increase the infiltration of cytotoxic CD8 T cells. PyMT mice with 32–64 mm^3^ mice were treated by i.p. injection with anti-PD-L1 (40 μg/mouse) or isotype control antibodies on days 3, 7, and 11, in combination with LG268 or control diet (Fig. 9a). Mice treated with the combination of LG268 plus anti-PD-L1 antibodies presented a significant increase in infiltration of CD8 T cells into the tumors (*p* = 0.04) and an increased ratio of CD8/CD4, CD25 (*p* = 0.03) (Fig. 9b). These changes correlated with an increase in the cytotoxic activity of CD8 that is observed as elevated cleaved caspase 3 staining^50^ in the combination group (Fig. 9c). No changes in other immune cell populations present in the tumors were observed; isotype control antibodies also had no effects (Supplementary Fig. 10).

No differences were observed in the final total tumor weights between the groups (Supplementary Fig. 10C). Mice treated with LG268 alone showed a significant decrease in spleen weight compared with mice in control diet, however this difference did not reflect a difference in the analyzed immune populations (Supplementary Fig. 11). In spleen and intact mammary glands only a decrease total CD3 T cells was observed between groups receiving LG268 alone or in combination with anti-PD-L1. No other differences in cell populations in intact mammary glands were observed (Supplementary Fig. 12).

## Discussion

In these studies, we explored the effects of LG268, an RXR agonist, as an immune modulator in the tumor microenvironment of two genetically and histologically distinct mouse models of breast cancer. A 5-day treatment with the potent rexinoid LG268 altered myeloid and T-cell populations in the tumor microenvironment of MMTV-Neu mice and significantly (*p* = 0.002) increased the expression of PD-L1. In contrast, the FDA-approved rexinoid bexarotene failed to show immune modulatory effects in this model. In addition, LG268 increased survival in the highly aggressive and chemoresistant PyMT mouse model (*p* = 0.018). When LG268 was used in combination with anti-PD-L1 antibodies, a significant (*p* = 0.05) increase in infiltration of CD8 cytotoxic T cells into tumors was observed. Our results provide a compelling rationale for the use of a RXR agonist such as LG268, but not bexarotene, in TNBC to increase the response to an anti-PD-L1 checkpoint inhibitor.

The only FDA-approved RXR agonist, bexarotene, has been tested in a clinical trial for treating breast cancer; unfortunately the clinical trial did not show conclusive benefits,^18^ and the immunologic effects were never explored.^51^ The expectation for the use of RXR agonists to treat solid tumors is that these small molecules will reduce cancer cell proliferation and induce apoptosis and/or differentiation of tumor cells.^25,52^ However, the immune regulatory effects of RXR are starting to be appreciated and explored in several contexts, such as viral infections, neurodegenerative diseases and cancer.^14,53–55^ LG268 binds more effectively to RXR than bexarotene (*K*_d_ 3 nm vs. 34 nm); whereas LG268 does not bind to the RAR receptor, bexarotene retains limited RAR binding.^56^ Moreover, LG268 is hypothesized to have higher immunologic efficacy than bexarotene,^14,16,57,58^ with bexarotene only showing activity when combined with vaccination.^57^ In addition, bexarotene failed to show any immune modulatory effects in cutaneous T-cell lymphoma.^58^

A growing body of evidence points to the importance of immune cell populations within the tumor and the likelihood of response to immunotherapy blockade;^59–61^ elevated levels of PD-L1 correlate with a high probability of response to immune checkpoint inhibitors^47^ and with a better prognosis in breast cancer patients.^48,62^ Favorable responses to immunotherapy are still low, with at best a 20% success rate, suggesting that new pharmacological strategies to increase response rates are required.^59^ Such strategies can come from the use of small molecules to extend the benefits of immunotherapy, to modulate the tumor microenvironment, to traffic CD8 cytotoxic T cells into the tumor, or to alter cytokine production.^63^ Data presented here strongly suggests that the rexinoid LG268 can be used to increase the responsiveness to anti-PD-L1 antibodies, which is especially important in TNBC, as a strong correlation between PD-L1 expression and survival has been demonstrated.^61^ In addition, results from the IMPassion130 trial^11^ suggest increased survival in metastatic breast cancer patients when treated with nanoparticle albumin-bound (nab)–paclitaxel and atezolizumab (anti-PD-L1) if immune cells within tumors are positive for the expression of PD-L1. Future studies will explore whether LG268 can increase response to PD-L1 checkpoint inhibitors in combination with conventional chemotherapy drugs.

Therapeutically, the most exciting findings reported here suggest that LG268 can reverse the immunosuppressive phenotype present in HER2-positive breast cancer and modulate the PD-1/PD-L1 pathway. Therefore, it would be clinically significant to test LG268 and novel RXR agonists, currently being developed in various laboratories, in solid tumors with low expression of PD-L1 and thus with anticipated poor responses to PD-L1 immune checkpoint blockade. This strategy might reverse immune tolerance and facilitate the development of effective combination therapies (including checkpoint inhibitors) with RXR agonists.

## Methods

### Drugs

LG268 was synthesized as described^21^ and provided by W. Lamph. Compound purity was >95%. Bexarotene was purchased from LC Laboratories (Lot BXR-108, purity >99%).

### Cell culture

RAW 264.7 mouse macrophage-like (ATCC) and E18-14C-27^24^ cells were cultured in Dulbecco’s Modified Eagle Medium (DMEM) supplemented with 10% fetal bovine serum (FBS). RAW 264.7 cells were cultured at 50,000/well in a six-well plate for 24 h to determine PD-L1 levels. E18-14C-27 cells were cultured for 72 h in the presence of LG268 or bexarotene for protein levels and flow cytometry. Conditioned media was collected from E18-14C-27 at 60–70% confluence at 24 h; media was collected and centrifuged at 900 rpm for 5 min to eliminate any cell debris.

THP-1 (ATCC) cells were cultured in Rosewell Park Memorial Institute (RPMI) supplemented with 5% FBS; SK-BR-3 (ATCC) cells were cultured in DMEM supplemented with 10% FBS. THP-1 cells were cultured at 50,000/well in a six-well plate for 24 h to determine PD-L1 levels with conditioned media from SK-BR-3 cells. Culturing SK-BR-3 cells at 60–70% confluence for 24 h generated conditioned media; media was then collected as described above. THP-1 cells were then plated at 100,000 cells/well with the conditioned media; LG268 was added simultaneously.

### Western blotting

For whole-cell lysates, cells treated with drugs were lysed in RIPA buffer (1 m Tris-Cl, pH 7.4, 0.5 m EDTA, 5 m NaCl, 1% triton-X, 25 mm deoxycholic acid, 0.1% SDS) containing protease inhibitors (PMSF, aprotinin, and leupeptin). For cytoplasmic vs. nuclear extracts, cells were incubated 15 min on ice with a cell lysis buffer (10 mm HEPES, 10 mm KCl, 1.5 mm MgCl_2_, 0.1 mm EGTA, 0.5% NP-40, 0.5 mm DTT) containing protease inhibitors (PMSF, aprotinin, and leupeptin). This extract was centrifuged and the remaining pellet (nuclear fraction) was incubated with the nuclear extraction buffer (10 mm HEPES, 420 mm NaCl, 1.5 mm MgCl_2_, 0.1 mm EGTA, 5% glycerol, 0.5 mm DTT) containing protease inhibitors (PMSF, aprotinin, and leupeptin). All blots derive from the same experiment and were processed in parallel.

Tumors or mammary glands were homogenized in EBC buffer (1 m Tris pH 8, 5 m NaCl) with the same protease inhibitors and 10% NP-40 and incubated on ice for 30 min. Protein concentrations were determined by the BCA assay (Sigma-Aldrich). Proteins were resolved by SDS-PAGE, transferred to a nitrocellulose membrane and analyzed with the following antibodies: PD-1 (1:1000, 5% milk, Abcam), CD206 (1:1000, 5% milk, Abcam); PD-L1 (4:1000, 5% milk, R&D); vinculin (1:4000, 5% BSA, Cell Signaling), p-STAT1 (1:1000, 5% BSA, Cell Signaling), p-STAT3 (1:1000, 5% BSA, Cell Signaling), p-STAT5 (1:1000, 5% BSA, Cell Signaling), H3 (1:4000, 5% BSA, Cell Signaling), β-tubulin (1:4000, 5% BSA, Cell Signaling), and mouse and rabbit secondary antibodies conjugated to horseradish peroxidase (HRP) (all from Cell Signaling, 1:1000 in 1% milk). ImageJ was used to quantify the immunoblots, and results were plotted and statistically analyzed using Prism 6. All images shown are representative of three independent experiments. All blots derive from the same experiment and were processed in parallel.

### In vivo experiments

All animal studies were done in accordance with protocols approved by the Institutional Animal Care and Use Committee at Michigan State University. MMTV-PyMT mice were obtained from Dr. Jeffrey Pollard (Albert Einstein College of Medicine, Bronx, NY) and were bred and genotyped as described.^29^ MMTV-Neu mice were obtained from Jackson Laboratory and mated as previously described;^28^ genotyping is not required as all females are MMTV-Neu positive. Four-week-old female PyMT or MMTV-Neu mice were fed 5002 rodent chow until tumors of 32–64 mm^3^ were detected; at that time point mice were randomized to either control powder chow or LG268 (100 mg/kg diet) mixed into powdered diet as described.^64^ During treatment, tumor volumes were measured twice weekly. Anti-PD-L1 and isotype control antibodies were purchased from Biolegend Go In vivo, and administered i.p. at 40 µg per mouse, on days 3, 7, and 11 of the 15 day regimen.

### Flow cytometry

One third of the tumor, mammary gland and spleen removed from MMTV-Neu or MMTV-PyMT female mice were minced separately and incubated in digestion media consisting of collagenase (300 U/ml, Sigma), dispase (1 U/ml, Worthington), and DNAse (2 U/ml, Calbiochem) for 30 min at 37 °C with stirring. Cells were then passed through a 40 µm cell strainer (BD Falcon), and red blood cells eliminated with lysis solution. Single cells were resuspended in a solution of PBS/0.5% BSA/0.1% azide and stained for 30 min at 4 °C with the following antibodies: CD45-VioGreen (30F11, Miltenyi, 10:100), Gr1-PE (RB6-8C5, Miltenyi, 10:100), CD11b-FITC (M1/70.15.11.5, Miltenyi, 10:100), CD19-APC (1D3/CD19, Biolegend, 1:100), B220-PerCP-Cy5.5 (RA3-6B2, Biolegend, 1:100), CD3-PE (145–2C11, Biolegend, 1:100), CD4-FITC (Gk1.5, Miltenyi, 10:100), CD8-APC (53–6.7, Biolegend, 1:100), CD25-PE.Cy7 (EBiosciences, 1:100) and 5 μg/ml anti-mouse CD16/CD32 antibody (Biolegend) to reduce antibody binding to Fc receptors. Propidium iodide staining was used to exclude dead cells. Cells were analyzed using a BD FACS ARIA (BD) with three laser sources (488 nm, 633 nm, 405 nm) and FlowJo x.10.0.7r2 software (Tree Star).

For PD-L1 staining, RAW 264.7 cells were plated at 50,000 cells/ml in six-well plates for 24 h before adding conditioned media from E18-14C-27 cells. RAW 264.7 cells were cultured for an additional 24 h with E18-14C-27 conditioned media and either LG268 or bexarotene. RAW 264.7 cells were collected and resuspended in a solution of PBS/0.5% BSA/0.1% azide and stained for 30 min at 4 °C with anti-PD-L1-APC (10 F.9G2, Biolegend, 1:100) and 5 μg/ml anti-mouse CD16/CD32 antibody (Biolegend) to reduce antibody binding to Fc receptors. Cells were analyzed using a BD Accuri C6 (BD) with two laser sources (488 nm and 640 nm) and FlowJo x.10.0.7r2 software (Tree Star). E18-14C-27 and THP-1 cells were stained and analyzed for the levels of PD-L1 following the same protocol. FlowJo x.10.0.7r2 software (Tree Star) was used to calculate the percentage of PD-L1-positive cells as MFI.

### Immunohistochemistry

One-third of the tumor, spleen, and one mammary were removed from female MMTV-Neu and PyMT mice and fixed in 10% phosphate-buffered formalin for 48 h, embedded in paraffin blocks, and sectioned (5–6 µm). Hydrogen peroxide was used to quench endogenous peroxidase activity. Sections were immunostained with antibodies raised against CD206 (1:200, Abcam), Gr1 (1:40, BM8, R&D), CD8 (1:40, 53–6.7, Biolegend), FOXP3 (1:50, FJK-16s, EBiosciences), PD-1 (1:100, EPR20665, Abcam), PD-L1 (1:50, MIH6, Abcam; R&D 1:50 for dual staining), cleaved caspase 3 (1:100, 5A1E, Cell Signaling), PCNA (1:200, sc-56, Santa Cruz Biothechonlogies), p-STAT1 (1:50, Cell Signaling), F4/80 (1:50, Abcam) and visualized with biotinylated anti-rabbit or anti-rat secondary antibodies (Cell Signaling or Vector Labs). Signal was detected using a DAB substrate (Cell Signaling) following the manufacturer’s recommendations. Sections were counterstained with hematoxylin (Vector Labs). Dual staining was performed with a Double Stain IHC kit using AP-rabbit and HRP-rat antibodies (Abcam, ab183285).

### CD4-FOXP3 T-cell treatment

From a single-cell suspension of splenocytes from a wild-type mouse, 10^7^ cells were incubated in 100 μL of Mojo Buffer (Biolegend) with 10 μL of pre-diluted Biotin-antibody cocktail mix for 15 min, followed by 10 μL of pre-diluted streptavidin nanobeads for an additional 15 min on ice (Biolegend MojoSort CD4 T-cell isolation kit). The mixture was then placed on the column in the magnetic separator and washed with 3 mL of Mojo Buffer. Isolated CD4 T cells were plated in a 24-well plate at 10^6^ ml/well in RPMI media supplemented with 10% FBS, anti-mouse CD28 (clone 37.51, 3 μg/mL, Biolegend), recombinant IL2 (5 ng/mL, Biolegend), and recombinant human TGFβ (5 ng/mL, R&D). Plates were coated with anti-mouse CD3ε (clone 145–2C11, 3 μg/mL, Biolegend) overnight at 4 °C. After 24 h, LG268 or bexarotene were added at 100 nm and CD4 T cells were cultured for an additional 4 days. On day 5 cells were collected and RNA was extracted using triZOL (Thermo Fisher Scientific) following the manufacturer’s instructions. Two micrograms of RNA were reverse transcribed, and 1 μl of complementary DNA from this reaction was added to 12.5 μl of Bio-Rad iQ SYBRGREEN Supermix (Hercules, CA), 1 μl of validated RT2 quantitative PCR (qPCR) FOXP3 (F: 5′-CTCGTCTGAAGGCAGAGTCA-3′; R: 5′-TGGCAGAGAGGTATTGAGGG-3′) or RLP13A (F: 5′-GTTGCCTTCACAGCGTA-3′; R: 5′-AGATGGCGGAGGTGCAG-3′) primers and DNAse-free water for real-time qPCR. All expression data were normalized using RLP13A as the housekeeping control.

### CD3 activation status

CD3 T cells were isolated as previously described for CD4, using a MojoSort Mouse CD3 T-cell isolation kit (Biolegend). Cells were plated in a previously coated 96-well plate (10^6^/mL) in RPMI supplemented with 10% FBS and LG268 at 100 and 1000 nm for 3 days. The plate was coated with CD3ε (clone 145–2C11, 5 μg/mL, Biolegend) overnight at 4 °C. Cells were collected and resuspended in a solution of Brilliant Stain Buffer (BD Horizon) and stained for 30 min at 4 °C with anti-CD3-Alexa Fluor 488, anti-CD4 Brilliant Violet 711, anti-CD8-Brilliant Violet 605, anti-CD25-PE, anti-CD44-PE-Cy5, anti-CD69-Brilliant Violet 421, anti-CD45-Alexa fluor 700 and anti-CD62L-APC (Biolegend, 1:100) and 5 μg/ml anti-mouse CD16/CD32 antibody (Biolegend) to reduce antibody binding to Fc receptors. Cells were analyzed using a BD FACS ARIA (BD) with three laser sources (488 nm, 633 nm, 405 nm) and FlowJo x.10.0.7r2 software (Tree Star).

### CD11b isolation

Tumors from MMTV-Neu female mice were minced separately and incubated in digestion media consisting of collagenase (300 U/ml, Sigma), dispase (1 U/ml, Worthington), and DNAse (2 U/ml, Calbiochem) for 30 min at 37 °C with stirring. Cells were then passed through a 40 µm cell strainer (BD Falcon). From a single-cell suspension 10^7^ cells were incubated in 100 μL of Mojo Buffer (Biolegend) with 10 μL of pre-diluted Biotin-antibody cocktail mix for 15 min, followed by 10 μL of pre-diluted streptavidin nanobeads for an additional 15 min on ice (CD11b cell isolation kit, Miltenyi). The mixture was then placed on the column in the magnetic separator and washed with 3 mL of Mojo Buffer. CD11b positive cells were then eluted from the column. Cells were lysed in RIPA buffer (1 m Tris-Cl, pH 7.4, 0.5 m EDTA, 5 m NaCl, 1% triton-X, 25 mm deoxycholic acid, 0.1% SDS) containing protease inhibitors (PMSF, aprotinin and leupeptin) for whole-cell lysates.

### Patient survival analysis

CD206 and PD-L1 were used for relapse-free survival analysis. For the meta-analysis cohort, we used aggregate data from KMPlot^41^ (http://www.kmplot.com) using auto-selection for best cutoff between the 25th and 75th percentiles. Owing to the retrospective nature of this study using only publicly available data, ethics approval for the study was not required.

### Statistical analysis

Unless noted, all experiments were repeated at least three times, and representative images are shown. Results are described as mean ± standard error of the mean. Data were analyzed by *t* test paired analysis with Welch’s correction for sets with two variables, or ordinary one-way analysis of variance with Dunnett’s multiple comparation test (Prism 6). All *p* values are two-sided; *p* < 0.05 was considered statistically significant.

### Reporting summary

Further information on research design is available in the Nature Research Reporting Summary linked to this article.

## Supplementary information

Supplementary Information

Reporting Summary Checklist

## Data Availability

The data generated and analyzed in this study are publicly available in the figshare repository here 10.6084/m9.figshare.9944942,^65^ and as part of the supplementary files. Human data can be accessed at https://kmplot.com/analysis/index.php?p=service&cancer=breast.
