# Supplementary material for: Retinoid X receptor agonist LG100268 modulates the immune microenvironment in preclinical breast cancer models
Source: NPJ Breast Cancer. 2019 Nov 1;5:39. doi: 10.1038/s41523-019-0135-5 (PMC6825145; doi:10.1038/s41523-019-0135-5)
Supplement: Supplementary file 1 — Supplementary Information [file 41523_2019_135_MOESM1_ESM.pdf]

Supplementary Information

Retinoid X receptor agonist LG100268 modulates the immune microenvironment in preclinical breast cancer models

Ana S. Leal[1], Kayla Zydeck[1], Sarah Carapellucci[1], Lyndsey A. Reich[1], Di Zhang[1], Jessica A. Moerland[1], Michael B. Sporn[2], Karen T. Liby[1*]

Affiliations:

[1]Michigan State University, Department of Pharmacology & Toxicology, East Lansing, MI

[2]Dartmouth/Geisel School of Medicine at Dartmouth, Department of Molecular and Systems Biology, Hanover, NH

*To whom correspondence should be addressed: Karen T. Liby, Michigan State University, Department of Pharmacology & Toxicology, B430 Life Science Building, 1355 Bogue Street, East Lansing, MI 48824. Phone: 517-884-8955; Fax: 517-353-8915; Email: liby.kare@msu.edu

Table of contents:

Supplementary Figure 1

Supplementary Figure 2

Supplementary Figure 3

Supplementary Figure 4

Supplementary Figure 5

Supplementary Figure 6

Supplementary Figure 7

Supplementary Figure 8

Supplementary Figure 9

Supplementary Figure 10

Supplementary Figure 11

Supplementary Figure 12

Supplementary Figure 13

Supplementary Figure 14

Supplementary Figure 15

Supplementary Figure 16

Supplementary Figure 17

Supplementary Figure 18

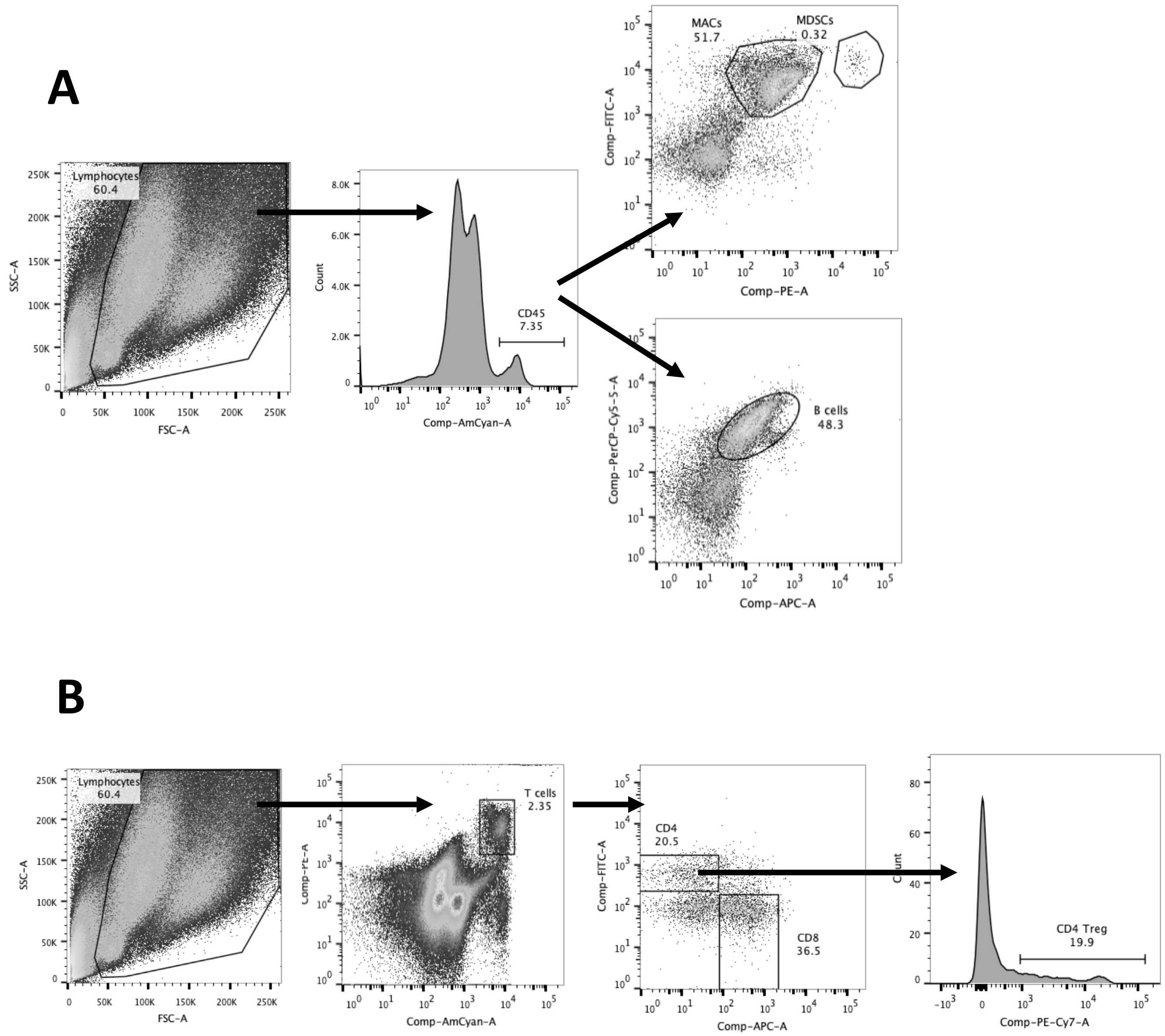

Supplementary Figure 1. Gating strategy used for flow cytometry analysis of myeloid and T cell populations. MACs= Tumor associated macrophages; MDSCs = Myeloid derived suppressor cells.

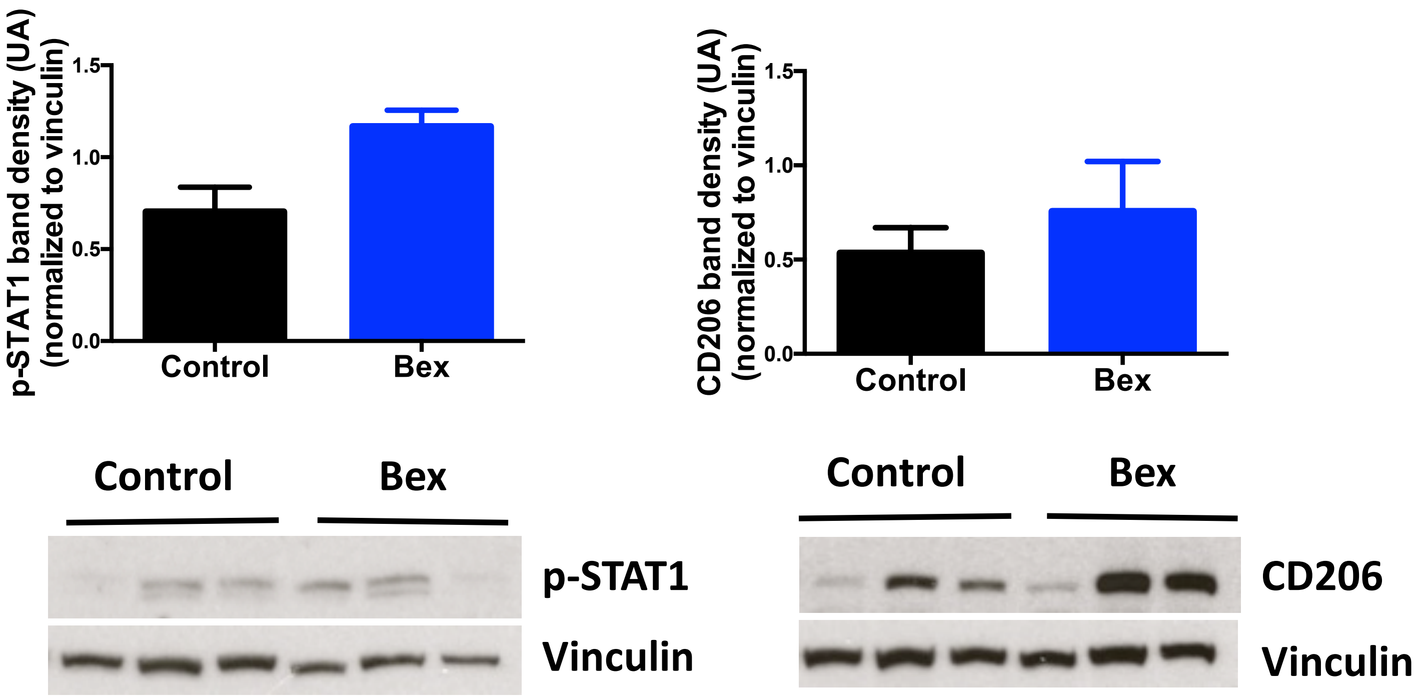

Supplementary Figure 2. Levels of p-STAT1 and CD206 in tumors of MMTV-Neu mice treated with Bexarotene (100 mg/kg diet) for 10 days. Levels of p-STAT1 and CD206 were evaluated in tumor lysates (n=3-4 mice/group). Quantification of the whole lysates is shown in the bar graphs, and a representative immunoblot is also shown. Bars represent average and error bars the standard error of the mean (SEM).

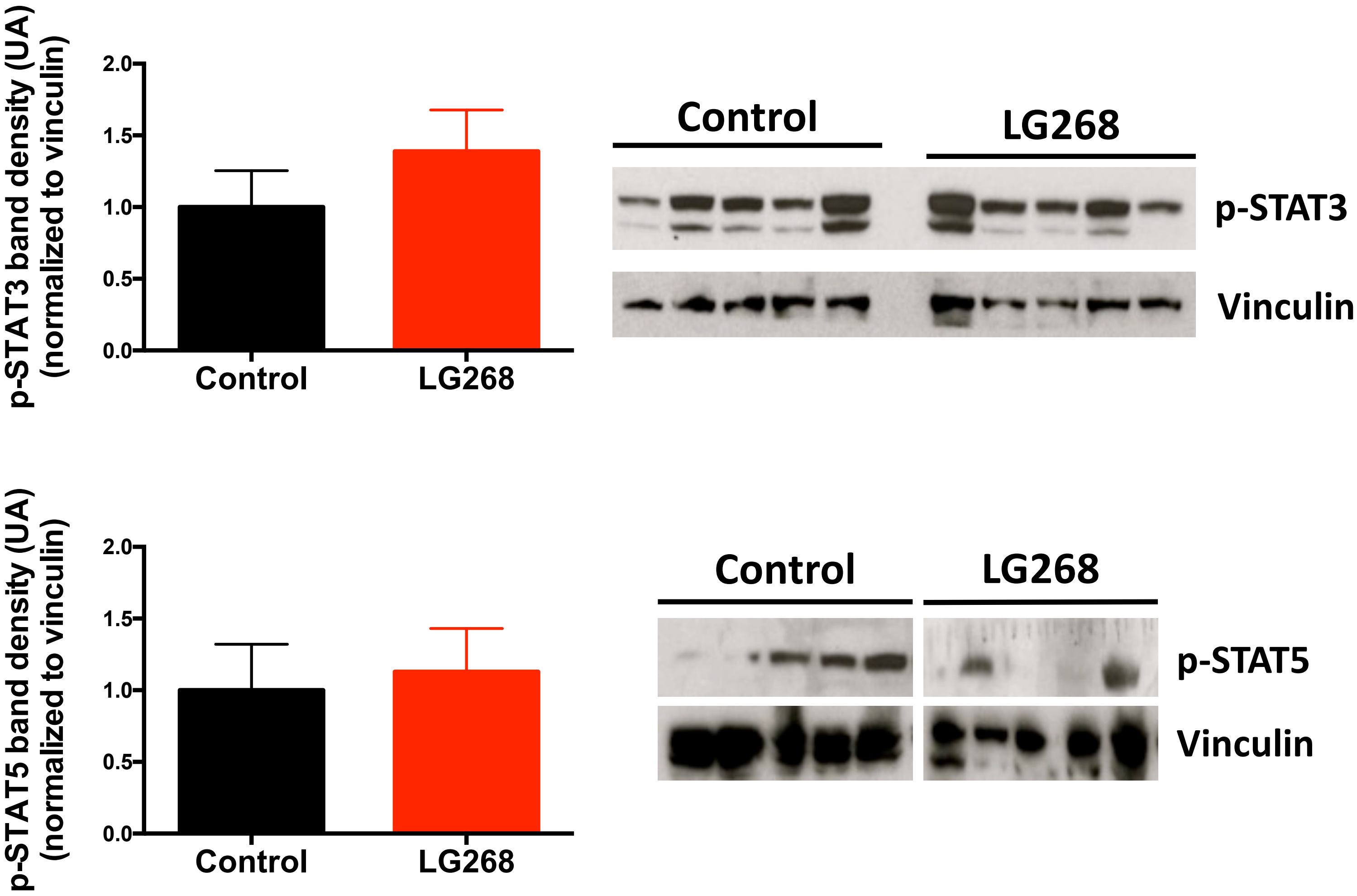

Supplementary Figure 3. Levels of p-STAT3 and p-STAT5 in tumor extracts of MMTV-Neu treated as shown in Figure 1 were analyzed by western blotting. Quantification of total protein levels in all samples is shown in the bar graph, and western blots of representative samples are shown. n=11, Bars represent average and error bars the standard error of the mean (SEM).

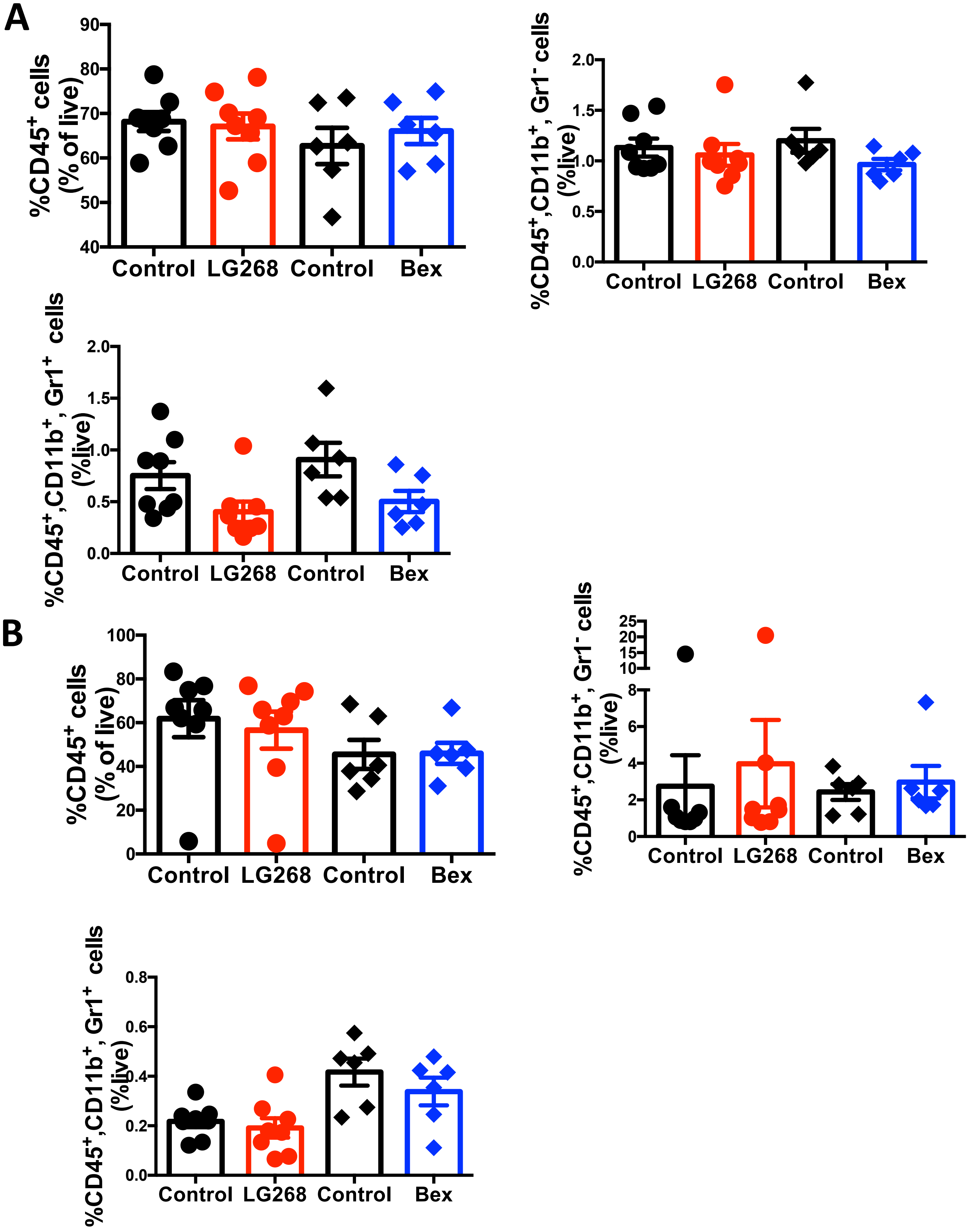

Supplementary Figure 4. Myeloid cell populations in the spleen (**A**) and in intact mammary gland (**B**) of MMTV-Neu mice treated as described in figure 1 were analyzed by flow cytometry. n=5-8, Bars represent average and error bars the standard error of the mean (SEM).

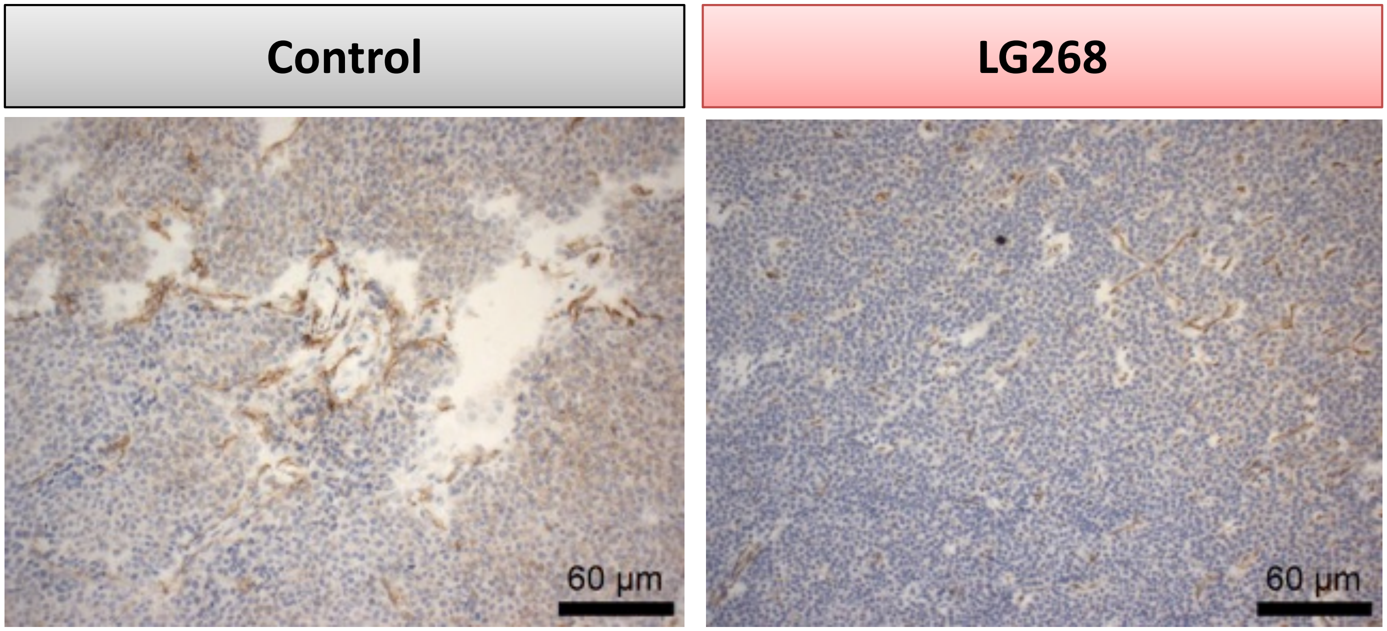

Supplementary Figure 5. LG268 treatment alters CD31 protein expression in endothelial cells. Expression of CD31 is altered in established tumors of MMTV-Neu mice treated with LG268 for 5 days, as shown by IHC. Vessels are smaller with a disorganized structure.

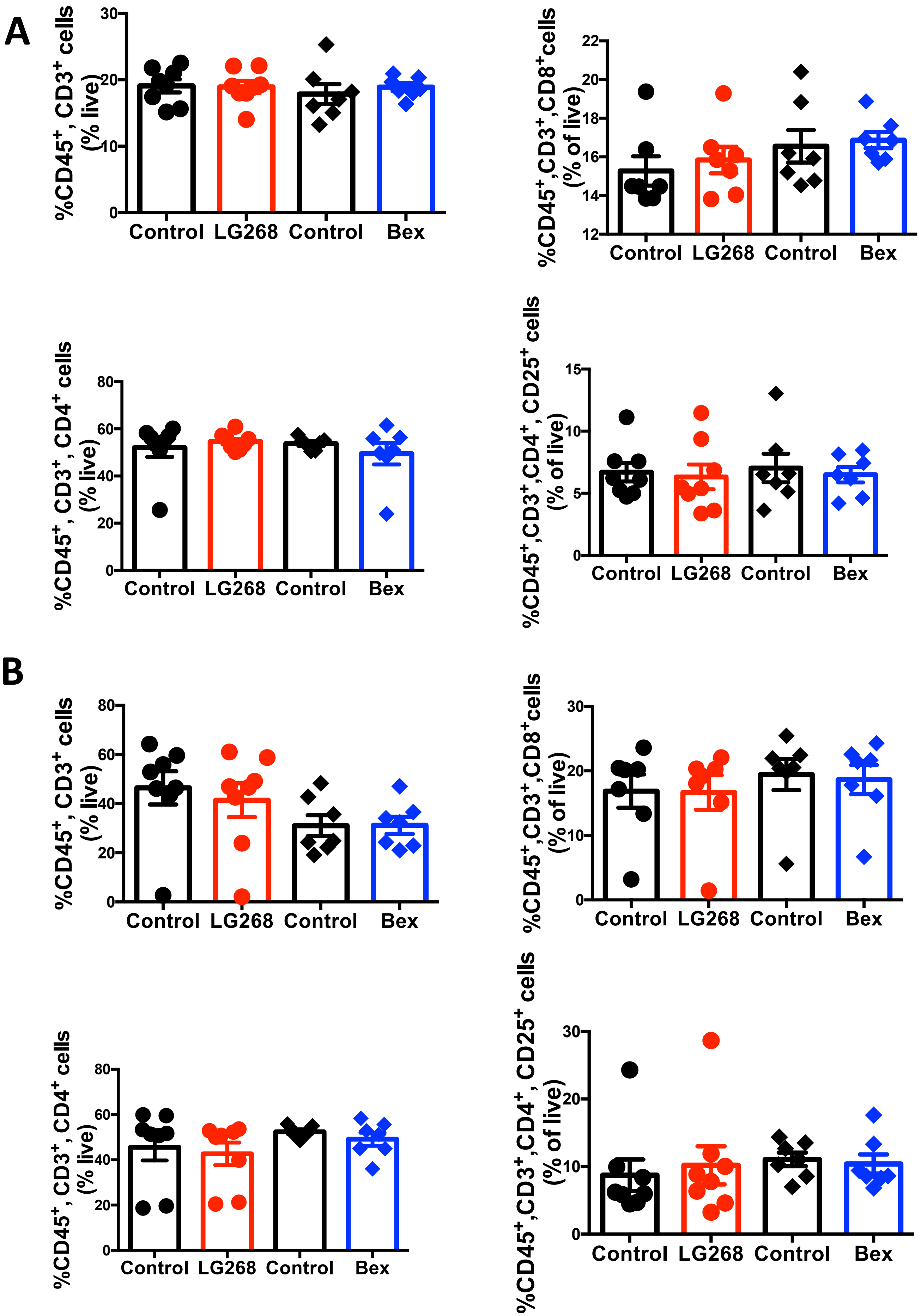

Supplementary Figure 6. T cell populations in the spleens (**A**) and in intact mammary glands (**B**) of MMTV-Neu mice treated as described in figure 1. n=5-8, Bars represent average and error bars the standard error of the mean (SEM).

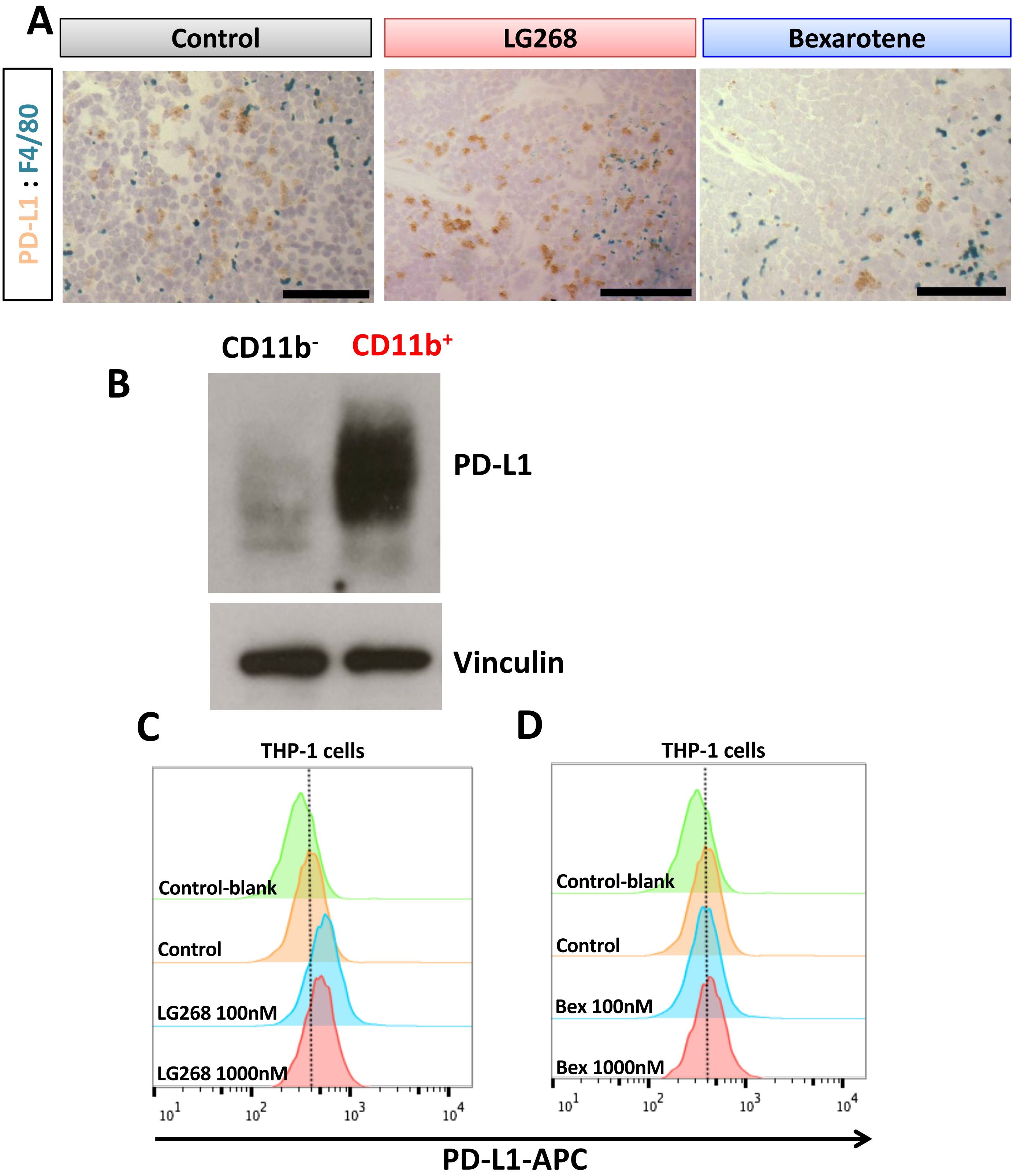

Supplementary Figure 7. Expression of PD-L1 in macrophages and THP-1 cells. (**A**) Dual Immunohistochemistry for PD-L1 (Brown), and F4/80 (green) in spleen of MMTV-Neu mice treated with LG268 or bexeratone. Scale bar represents 60 µm. (**B**) CD11b cells were isolated from a tumor of a MMTV-Neu mouse using positive magnetic beads. Levels of PD-L1 were determined by immunobloting**.** THP-1 human macrophages stimulated with conditioned media from human SK-BR-3 breast cancer cells and treated with LG268 (**C**) or bexarotene (**D**) were analyzed by flow cytometry to determine levels of PD-L1.

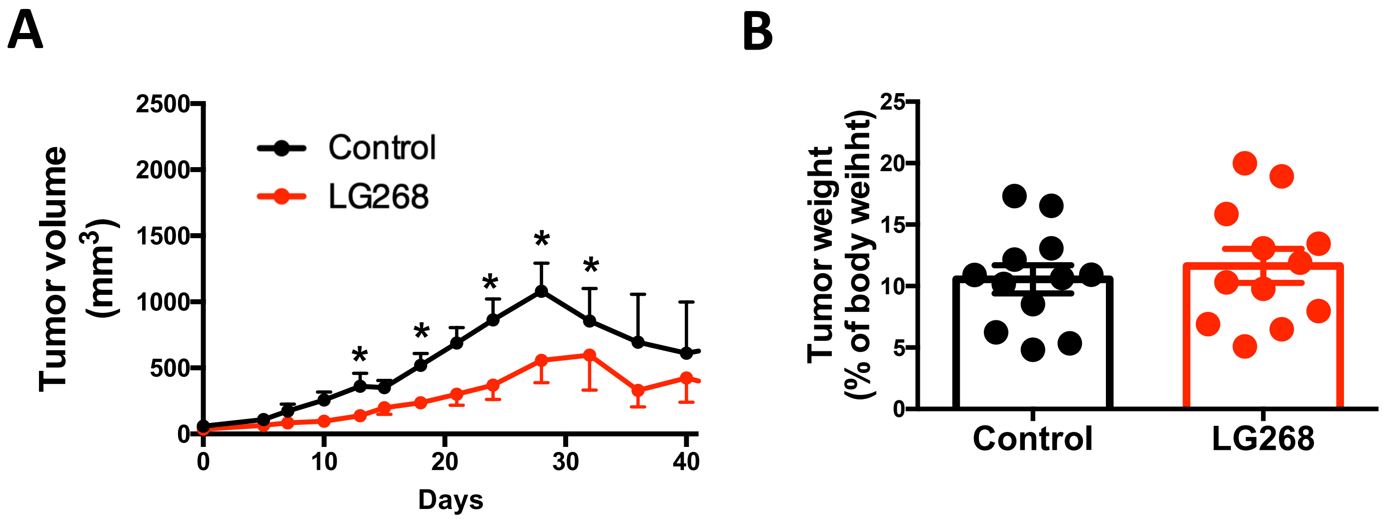

Supplementary Figure 8. LG268 reduces tumor burden in PyMT mice. PyMT mice with mammary tumor(s) with an initial volume of 32-64 mm^3 were treated with control diet or LG268 (100 mg/kg diet). (**A**) Combined tumor volume of all mice treated with control diet or LG268 (100 mg/kg diet) for up to 40 days. (**B**) Tumor weights from the end of the study (n=12 mice/group). * $p < 0.05$ vs control. Bars represent average and error bars the standard error of the mean (SEM).

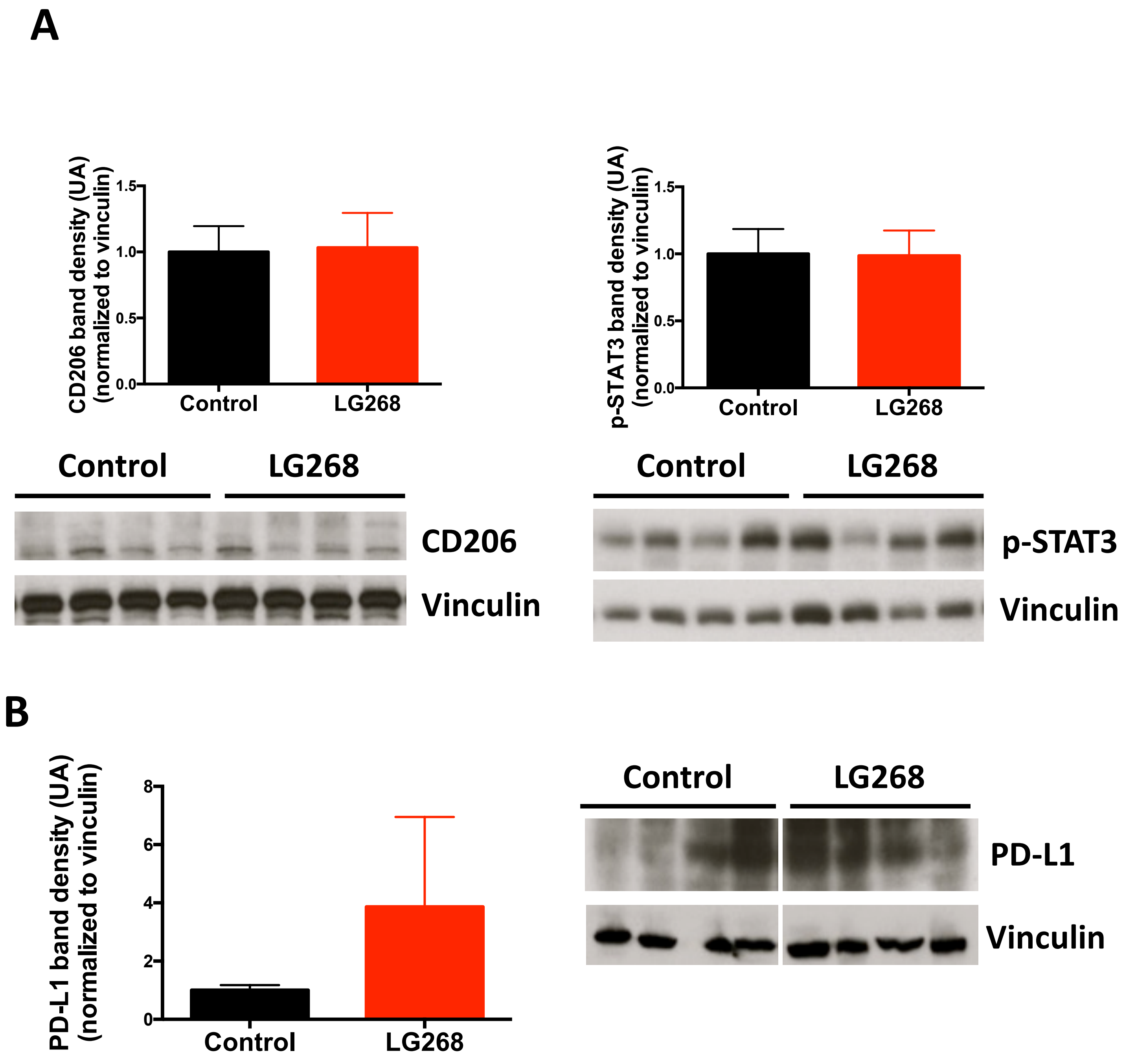

Supplementary Figure 9. Levels of protein in tumor extracts at the end point (**A**) or for 14 days (**B**) for PyMT mice treated with control diet or LG268 (100 mg/kg diet). Protein levels were determined by western blot; 4 mice per cohort are shown. n=7-12, Bars represent average and error bars the standard error of the mean (SEM).

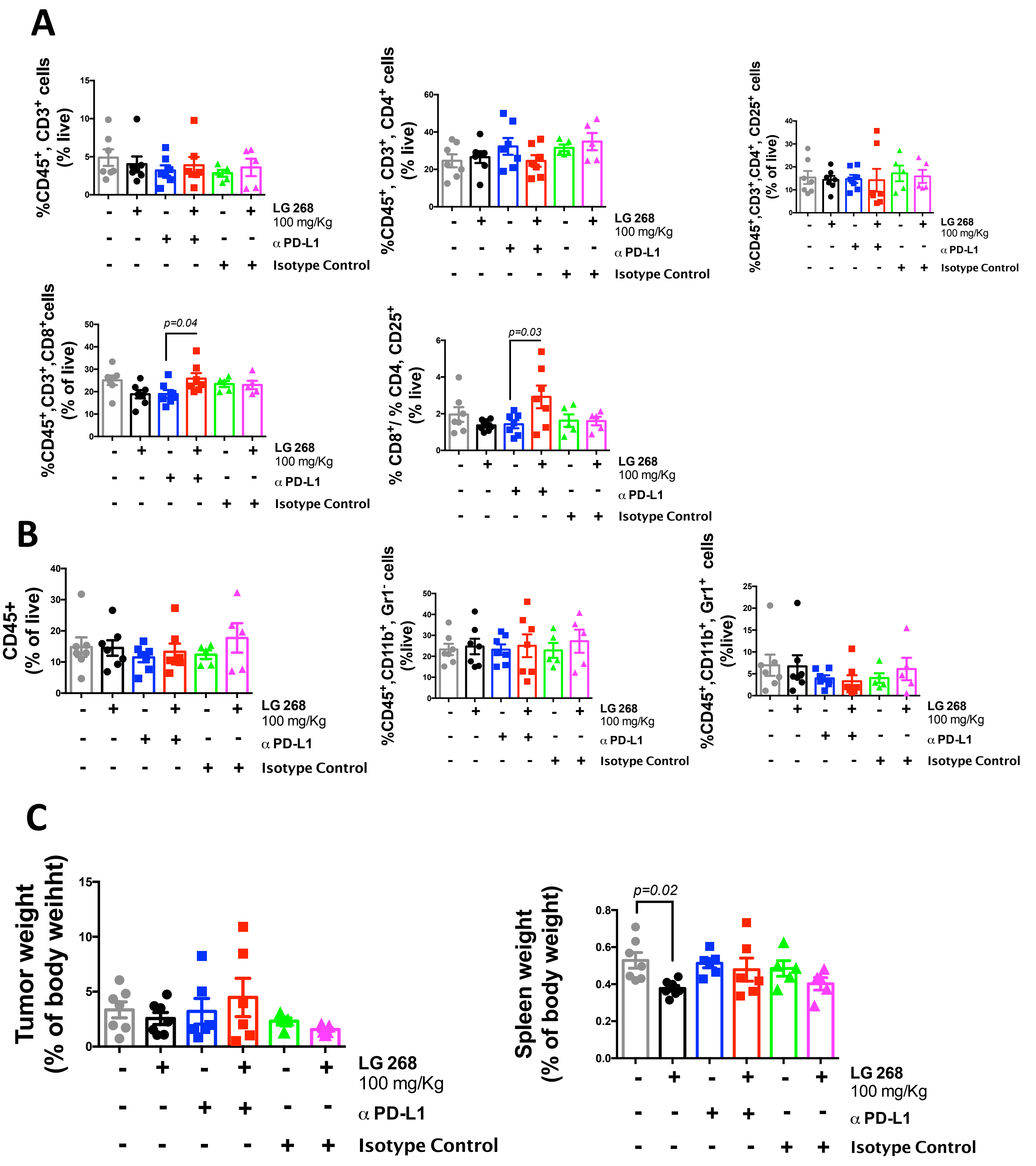

Supplementary Figure 10. (A) Immune cell infiltration of T cells in tumors was analyzed by flow cytometry in the 6 groups of PyMT mice. **(B) Myeloid cell populations** in the tumors of PyMT mice, as measured by flow cytometry, 15 days after the indicated treatments shown in figure 9. (**C**) Effects of treatment for 15 days on PyMT mice on tumor and spleen weights. n=5-7, Bars represent average and error bars the standard error of the mean (SEM).

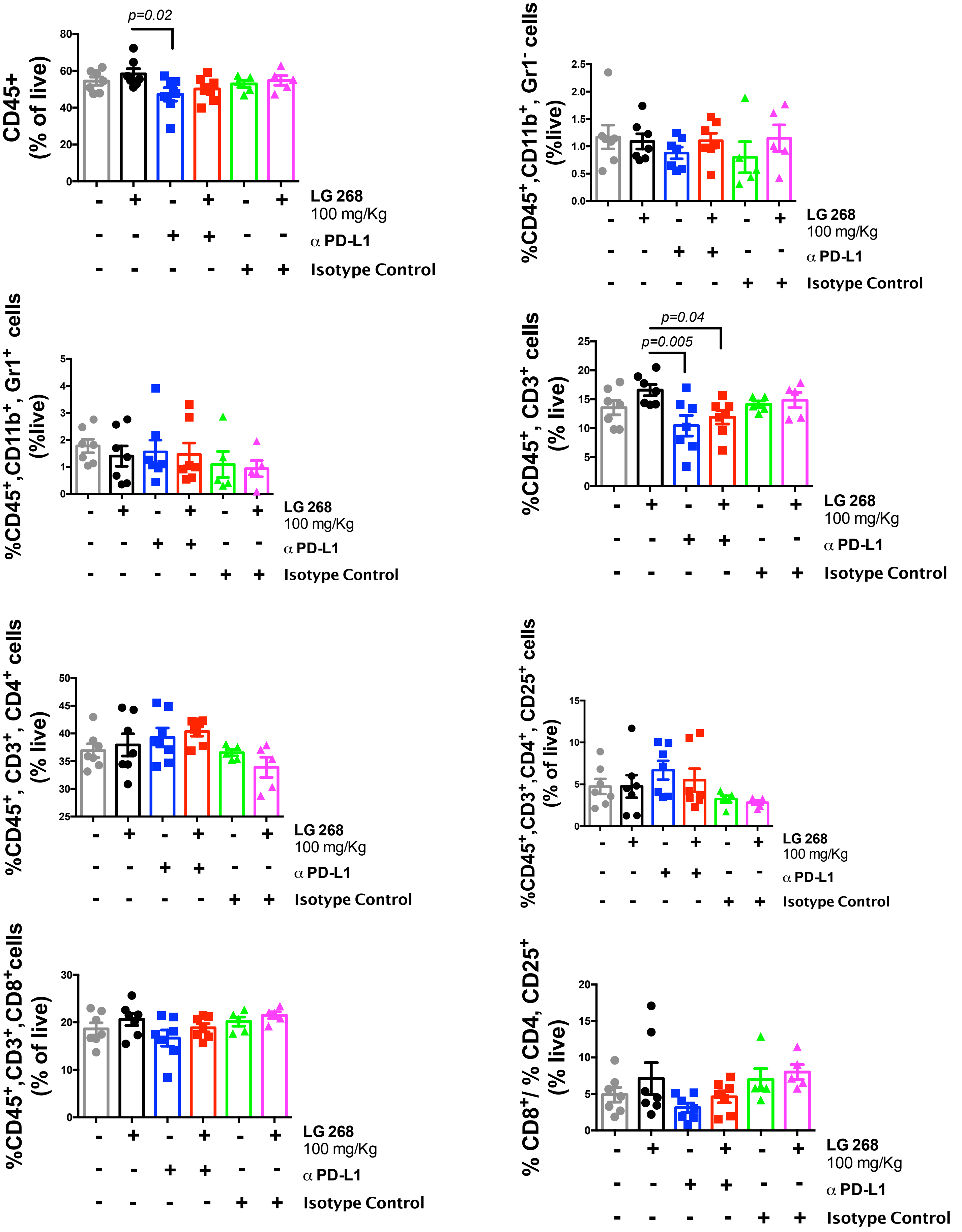

Supplementary Figure 11. Cell populations in the spleen of PyMT mice treated for 15 days were analyzed by flow cytometry. Treatment followed the regimen shown in figure 9. n=5-7, Bars represent average and error bars the standard error of the mean (SEM).

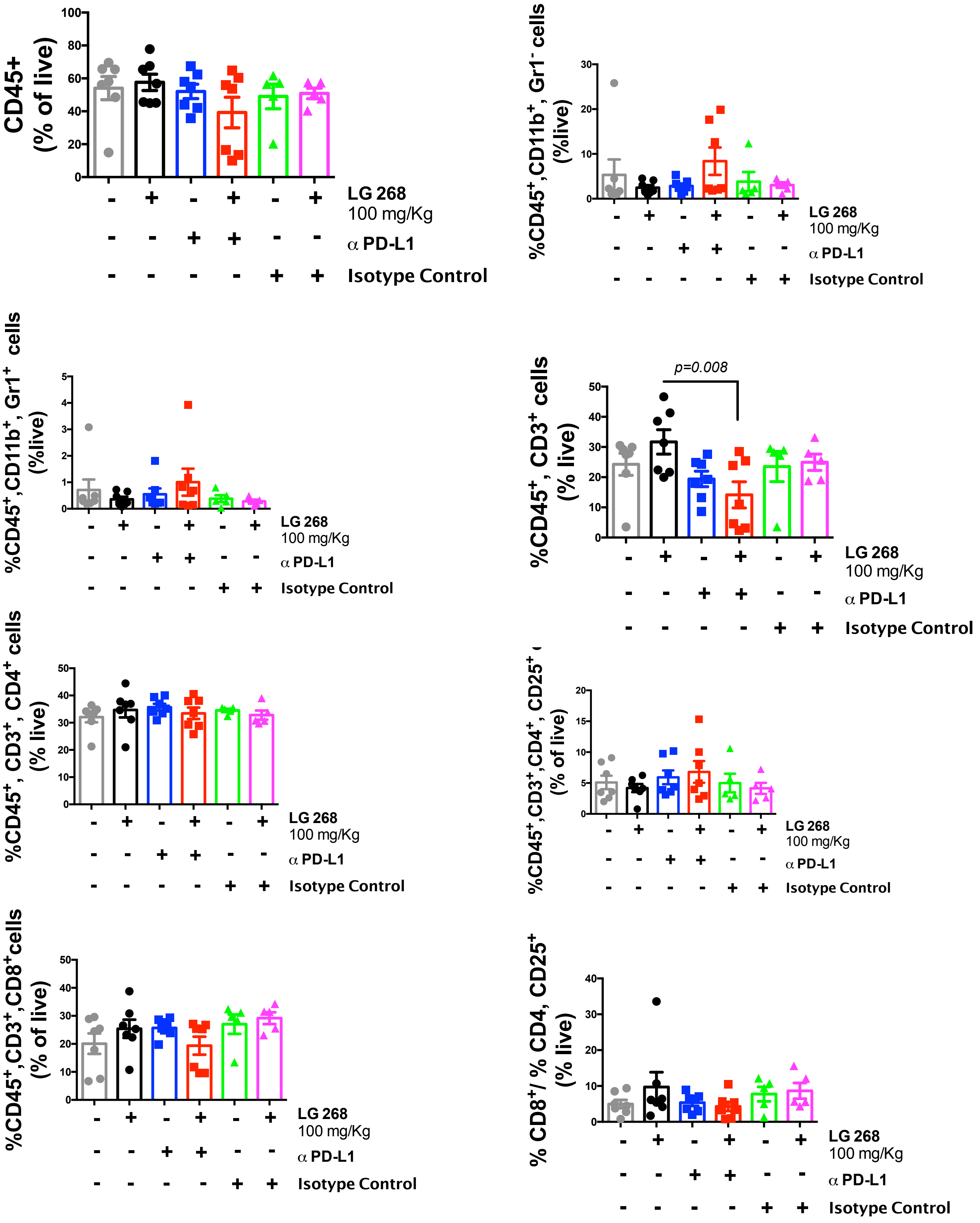

Supplementary Figure 12. Cell populations in intact mammary glands of PyMT mice treated for 15 days. Treatment followed the regimen shown in figure 9. n=5-7, Bars represent average and error bars the standard error of the mean (SEM).

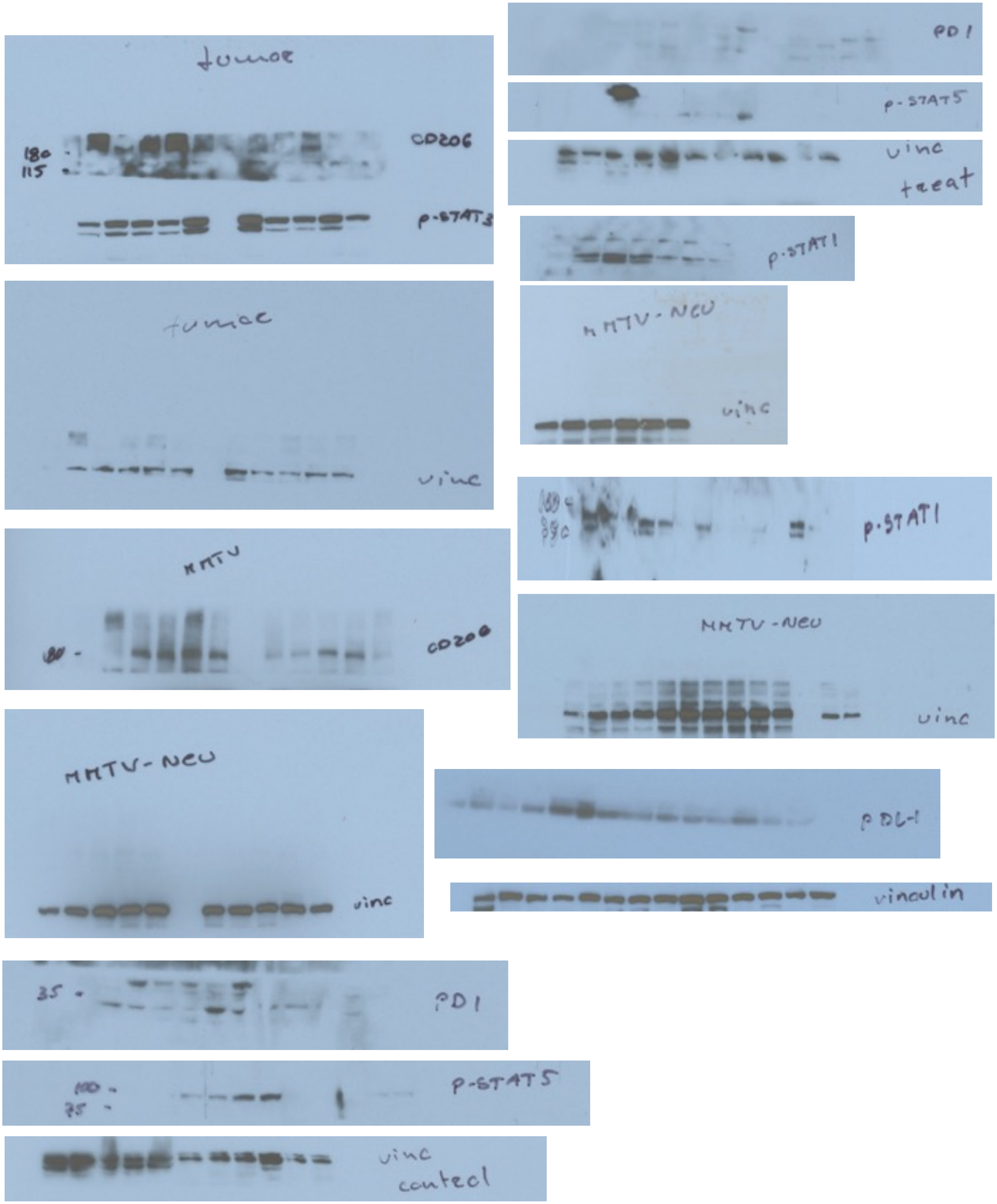

Supplementary Figure 13. Immunoblots used for the quantification of CD206, PD-L1, PD-1, p-STAT1, p-STAT5 and p-STAT3 in tumors of MMTV-Neu mice treated with LG268 and respective controls.

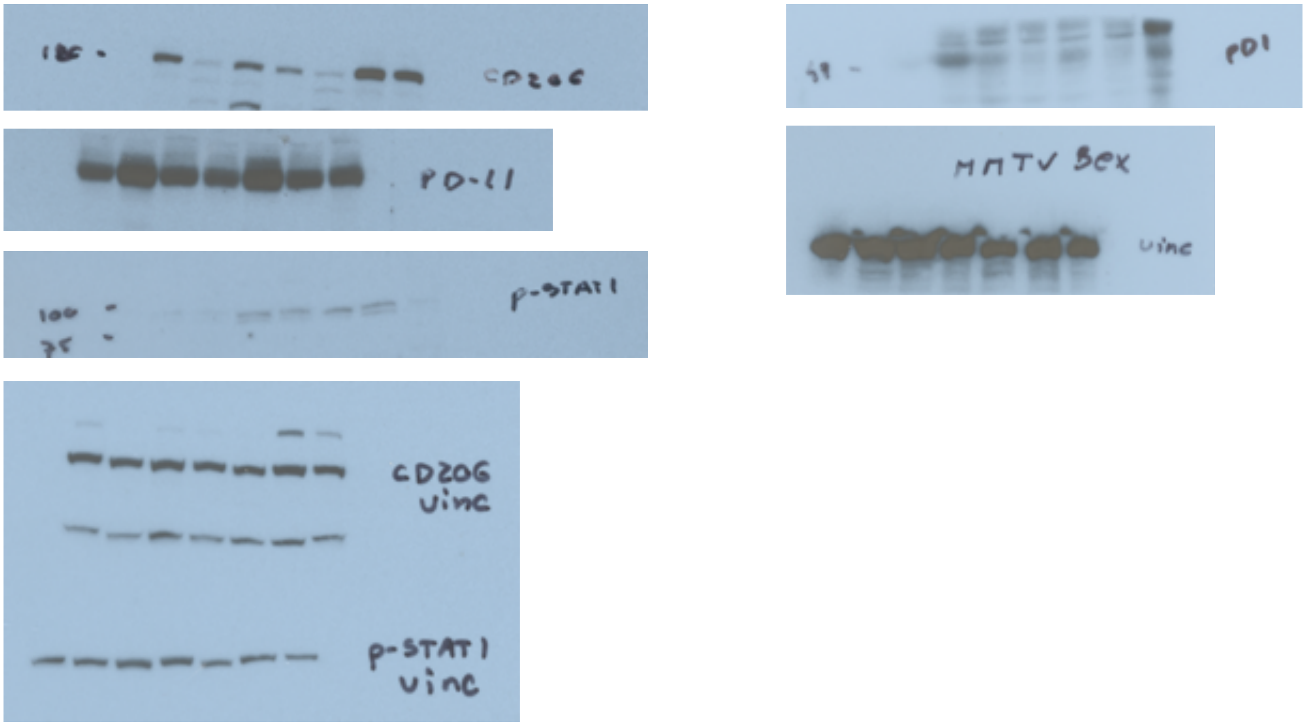

Supplementary Figure 14. Immunoblots used for the quantification of CD206, PD-L1, PD-1, p-STAT1, in tumors of MMTV-Neu mice treated with Bexarotene and respective controls.

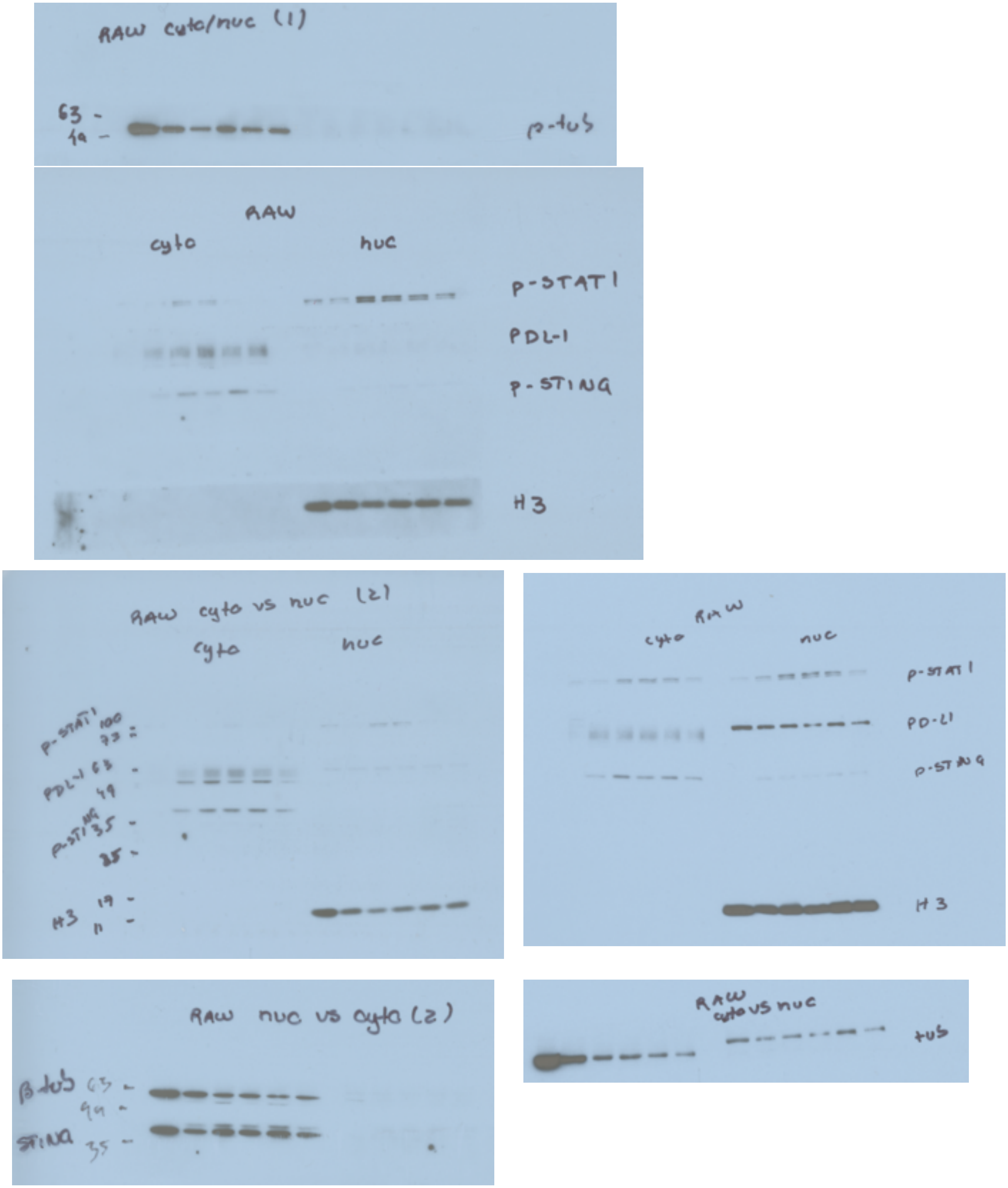

Supplementary Figure 15. Immunoblots of 3 independent experiments for figure 5E.

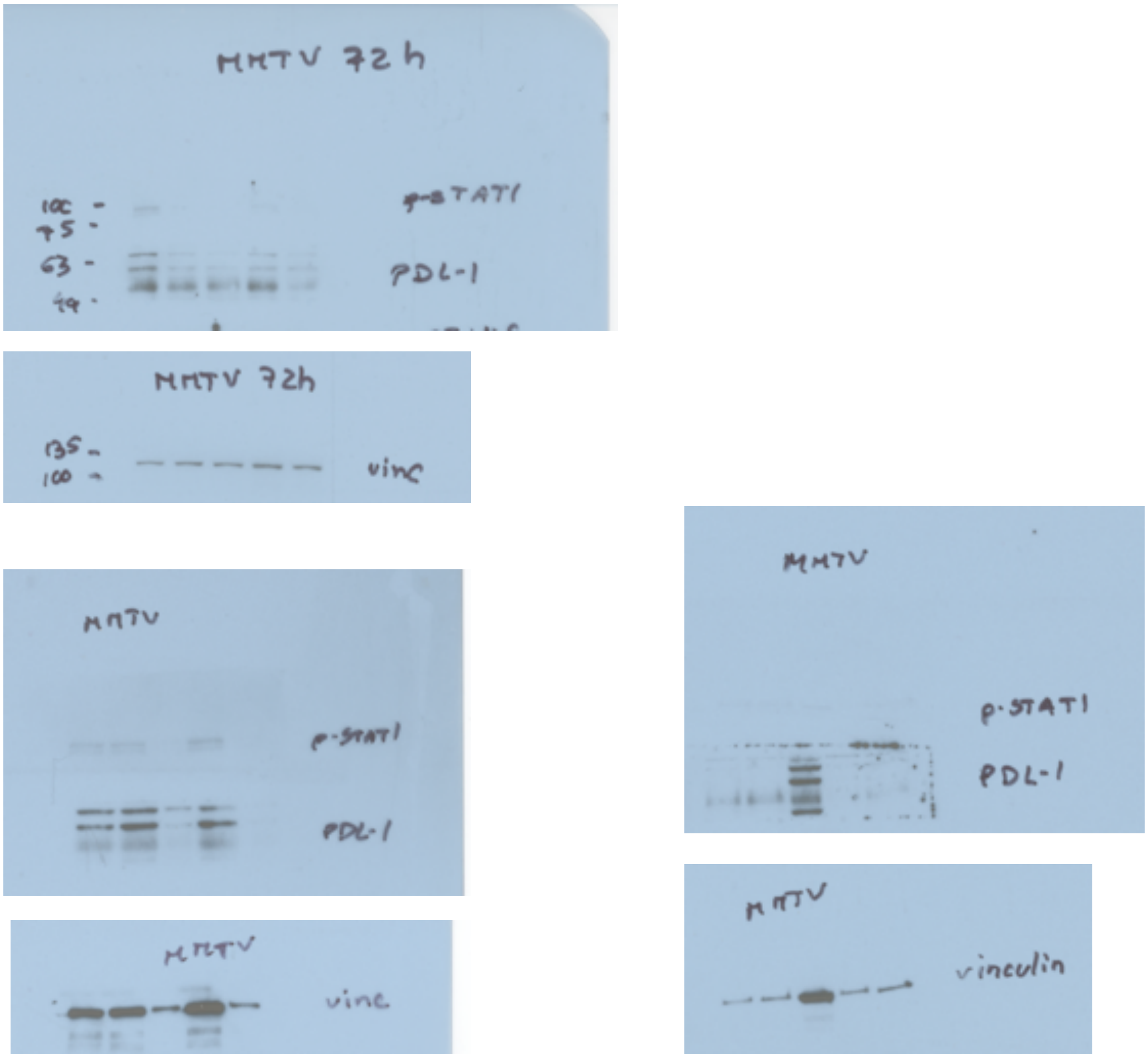

Supplementary Figure 16. Immunoblots of 3 independent experiments for figure 5I.

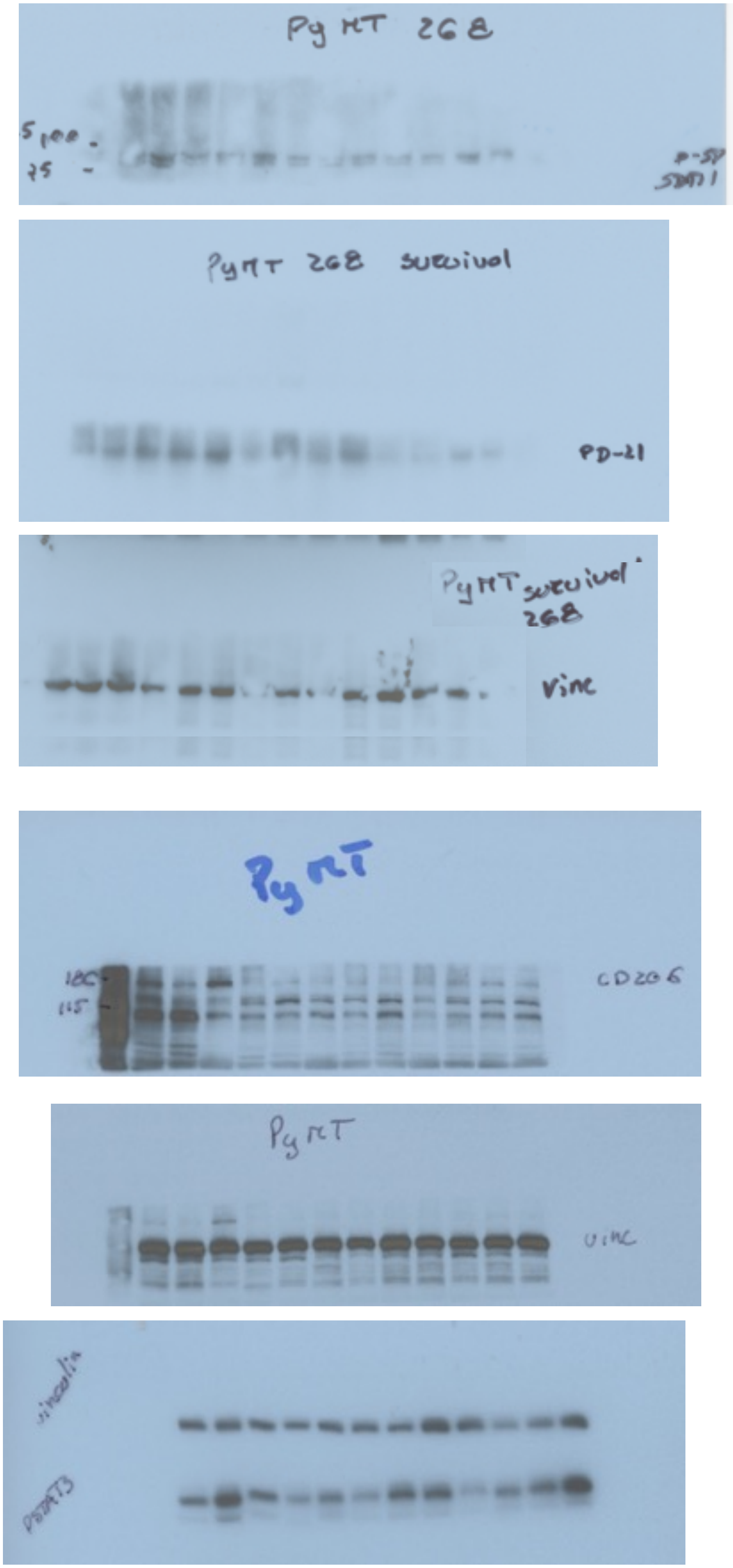

Supplementary Figure 17. Immunoblots used for the quantification of CD206, PD-L1, p-STAT1, and p-STAT3 in tumors of PyMT mice treated with LG268 and respective controls.

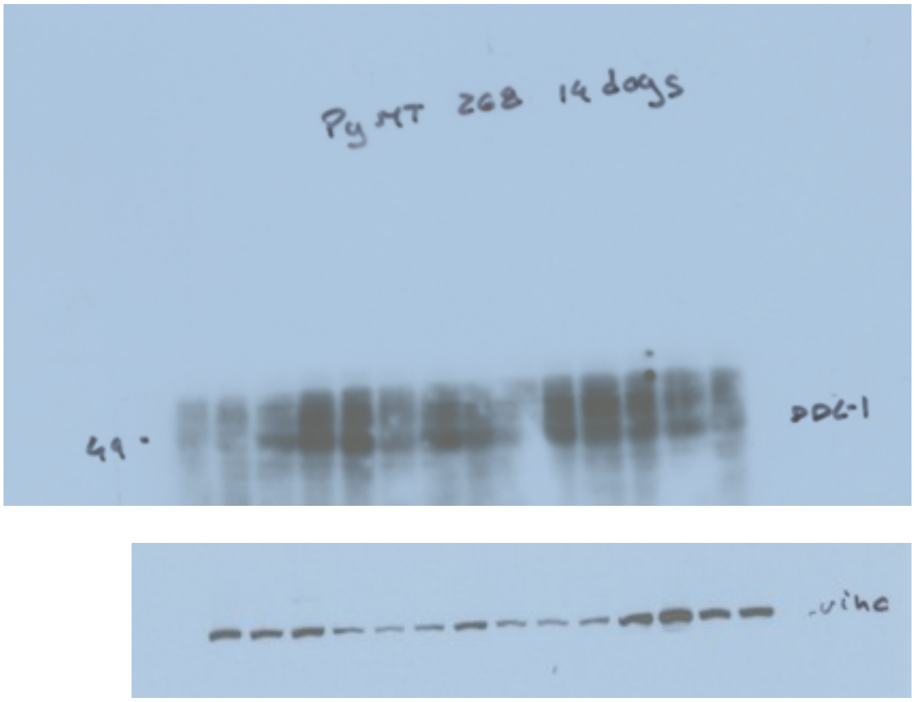

Supplementary Figure 18. Immunoblots used for the quantification of PD-L1 in tumors of PyMT mice treated with LG268 for 14 days and respective controls.
